# Scalable Bayesian Inference for Coupled Hidden Markov and Semi-Markov Models

**DOI:** 10.1080/10618600.2019.1654880

**Published:** 2019-09-18

**Authors:** Panayiota Touloupou, Bärbel Finkenstädt, Simon E. F. Spencer

**Affiliations:** Department of Statistics, University of Warwick, Coventry, UK

**Keywords:** Coupled hidden Markov model, Data augmentation, Epidemics, Forward–backward algorithm, Markov chain Monte Carlo methods

## Abstract

Bayesian inference for coupled hidden Markov models frequently relies on data augmentation techniques for imputation of the hidden state processes. Considerable progress has been made on developing such techniques, mainly using Markov chain Monte Carlo (MCMC) methods. However, as the dimensionality and complexity of the hidden processes increase some of these methods become inefficient, either because they produce MCMC chains with high autocorrelation or because they become computationally intractable. Motivated by this fact we developed a novel MCMC algorithm, which is a modification of the forward filtering backward sampling algorithm, that achieves a good balance between computation and mixing properties, and thus can be used to analyze models with large numbers of hidden chains. Even though our approach is developed under the assumption of a Markovian model, we show how this assumption can be relaxed leading to minor modifications in the algorithm. Our approach is particularly well suited to epidemic models, where the hidden Markov chains represent the infection status of an individual through time. The performance of our method is assessed on simulated data on epidemic models for the spread of *Escherichia coli* O157:H7 in cattle. Supplementary materials for this article are available online.

## 1 Introduction

Hidden Markov models (HMMs) are among the most widely used approaches for modeling time series data, when it can be assumed that the observed data are indicative of some underlying hidden process. In the basic HMM, a single variable represents the state of the system at any time. However, many interesting systems are composed of multiple interacting processes, and various extended HMMs have been proposed to solve coupled, multiple chain problems. These extensions typically factor the HMM state into a collection of state variables. We focus on coupled hidden Markov models (CHMMs; Brand 1997) to capture the interactions, where the current state of a chain depends on the previous state of all the chains. This structure implies that the state space of the complete hidden process grows exponentially with respect to the number of chains and thus exact inference quickly becomes computationally intractable.

Epidemiological data from infectious disease studies are often gathered longitudinally, where the same group of individuals are sampled through time. Inferences for this type of data are complicated by the fact that the data are usually incomplete, in the sense that the times of acquiring and clearing infection are not directly observed. CHMMs provide a natural way to model the transmission dynamics of an infectious disease, where each chain represents the hidden infection status of an individual and the coupling between chains accounts for infections. Another advantage of this approach is the ability to account for imperfect diagnostic tests, by assuming that the observed data are noisy measurements of a true hidden epidemic process.

The inference problem for CHMMs usually includes both hidden state and parameter estimation. Early literature on the topic focused on maximum likelihood estimation, achieved using an expectation-maximization (EM) algorithm. Several variations of the CHMM were proposed (Brand, Oliver, and Pentland 1997; Saul and Jordan 1999; Zhong and Ghosh 2002) for which inference using this approach becomes more tractable. The second class of methods consists of Markov chain Monte Carlo (MCMC) approaches. One considerable challenge concerns the imputation of the hidden states conditional on the observed data and model parameters, and many techniques have been proposed. The most popular approach to exact Monte Carlo inference is achieved by converting the CHMM into an equivalent single HMM and applying the standard forward filtering backward sampling (FFBS) algorithm (Carter and Kohn 1994; Chib 1996). However, even though implementation of FFBS is quite efficient for HMMs with a moderately large number of states, it can be computationally prohibitive for CHMMs with only a small number of chains. As a result, several alternative methods have been suggested including conditional single-site (Dong, Pentland, and Heller 2012) or block updates designed specifically for epidemic models (Spencer et al. 2015). While these methods are computationally less demanding than the FFBS, they typically produce highly correlated samples.

In this article, we develop two novel algorithms for updating the hidden chains within a MCMC algorithm. In particular, we propose a Gibbs sampling algorithm for the CHMM which is based on simulating from the full conditional distribution of a single chain, and a Metropolis–Hastings (MH) algorithm where the proposal is an approximation of the full conditional distribution. Section 3 describes the new algorithms and compares them with existing literature. In Section 4, we put CHMMs in the context of modelling the spread of infectious diseases, illustrating the efficiency and computational requirements of each algorithm using simulation studies. We subsequently describe how the proposed method can be extended to coupled hidden semi-Markov models (CHSMMs), where the hidden process persists in the same state for some non-Markov duration. In Section 5, we conclude with some discussion and possible extensions.

## 2 Coupled Hidden Markov Models and Notation

A CHMM is a collection of many HMMs, which are coupled through some temporal dependency structure of the hidden states. There are two conditional independence assumptions made about the observations and states. As in HMMs, in the CHMM each observation is independent of all other states and observations given the value of the hidden state. The difference with HMMs is that in the CHMM one hidden state is not only dependent on its own previous state, but also on the previous state of all other chains. The latter dependence constitutes the interaction between the multiple chains.

**Fig. 1:**
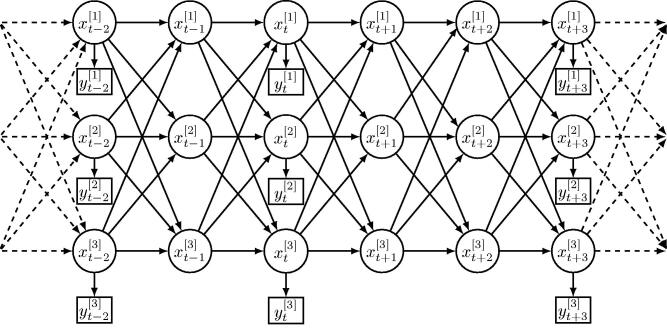
An example of a coupled hidden Markov model represented as a dynamic Bayesian network, with three hidden chains (*C* = 3) and possibly several missing observations (here at t−1,t+1,t+2). Circle nodes denote hidden states, square nodes denote observations, and the arrows between nodes reflect the probabilistic dependencies between random variables.

The coupling structure of a CHMM is shown in Figure 1. More formally, we use $X_{t}^{\left[c\right]}$ to denote the hidden state variable of chain c∈{1,2,…,C} at time t∈{1,2,…,T} with a finite set of possible states. For simplicity, we assume that all chains share the same set of possible states, noting that the method can be trivially extended to the more general case where chains do not share the same state space. Therefore, we assume that $X_{t}^{\left[c\right]}\in \Omega =\{s_{1},s_{2},\ldots ,s_{N}\},N\geq 1$. We consider nonhomogeneous Markov chains in which the transition probabilities depend on time given by

(1) $$P\left(X_{t}^{\left[c\right]}=j|X_{t-1}^{\left[c\right]}=i,X_{t-1}^{\left[-c\right]},\theta \right),$$

for all i,j∈Ω, where $X_{t-1}^{\left[-c\right]}$ denotes $\left(X_{t-1}^{\left[1\right]},X_{t-1}^{\left[2\right]},\ldots ,X_{t-1}^{\left[C\right]}\right)$ with $X_{t-1}^{\left[c\right]}$ removed and θ is the parameter vector of the CHMM model. To fully define the distribution of the hidden states, an initial distribution for $X_{1}^{\left[1:C\right]}=(X_{1}^{\left[1\right]},\ldots ,X_{1}^{\left[C\right]})$ must also be specified.

The state of each chain is not directly observable. As in HMMs, an observation $Y_{t}^{\left[c\right]}$ is associated with the unobserved state $X_{t}^{\left[c\right]}$. The relation between $X_{t}^{\left[c\right]}$ and $Y_{t}^{\left[c\right]}$ will differ depending on the application and $Y_{t}^{\left[c\right]}$ may be either discrete or continuous. Conditional on θ and $X_{t}^{\left[c\right]}=i$ denote the density or probability mass function of $Y_{t}^{\left[c\right]}$ by

(2) $$\pi \left(Y_{t}^{\left[c\right]}=y_{t}^{\left[c\right]}|X_{t}^{\left[c\right]}=i,\theta \right)=f_{i}\left(y_{t}^{\left[c\right]}|\theta \right),\;i\in \Omega .$$

If there is no observation at time *t* for chain *c* then $y_{t}^{\left[c\right]}$ is empty due to missing data and we set $f_{i}\left(y_{t}^{\left[c\right]}|\theta \right)=1$.

## 3 Bayesian Analysis and MCMC Methods

### 3.1 Overview

One considerable challenge on estimating CHMMs is that the likelihood function of the observed data given the model parameters is computationally intractable for even moderate numbers of states or interacting chains. This is because the likelihood involves summation over all possible configurations of the hidden state variables, where the dependencies within the state process make this calculation highly involved. One of the most popular methods adopted to overcome this issue is the use of data augmentation, in which the hidden states are treated as additional parameters and are imputed from the data. In the Bayesian framework this is facilitated by the use of MCMC algorithms, which enable the imputation of the hidden states and parameter estimation to be performed simultaneously.

For a prior π(θ), this approach yields a joint posterior density for the unobserved states and the model parameters that is known up to proportionality,

(3) $$\begin{array}{c} \pi \left(\theta ,X_{1:T}^{\left[1:C\right]}|Y_{1:T}^{\left[1:C\right]}\right)\propto \pi (\theta )P\left(X_{1}^{\left[1:C\right]}|\theta \right)\\ \;\times (\prod_{t=2}^{T}\prod_{c=1}^{C}P\left(X_{t}^{\left[c\right]}|X_{t-1}^{\left[c\right]},X_{t-1}^{\left[-c\right]},\theta \right))\\ \;\times (\prod_{t=1}^{T}\prod_{c=1}^{C}\pi \left(Y_{t}^{\left[c\right]}|X_{t}^{\left[c\right]},\theta \right)), \end{array}$$

where we adopt the following conventions $X_{t}^{\left[1:C\right]}=(X_{t}^{\left[1\right]},X_{t}^{\left[2\right]},\ldots ,X_{t}^{\left[C\right]})$ and $X_{1:t}^{\left[1:C\right]}=(X_{1}^{\left[1:C\right]},X_{2}^{\left[1:C\right]},\ldots ,X_{t}^{\left[1:C\right]})$ with similar notation applied to $Y_{t}^{\left[1:C\right]}$ and $Y_{1:t}^{\left[1:C\right]}$.

Samples from the joint posterior of the model parameters and the hidden states are generated by iteratively alternating between updating θ, conditional on the current values of $X_{1:T}^{\left[1:C\right]}$, and $X_{1:T}^{\left[1:C\right]}$ conditional on θ. The main interest in this article lies in the update of the hidden process which is the most computational demanding part. Before discussing the details of our new approaches in Section 3.3, we first briefly describe the standard algorithms for the CHMMs within this framework.

### 3.2 Existing Methods

**Fig. 2:**
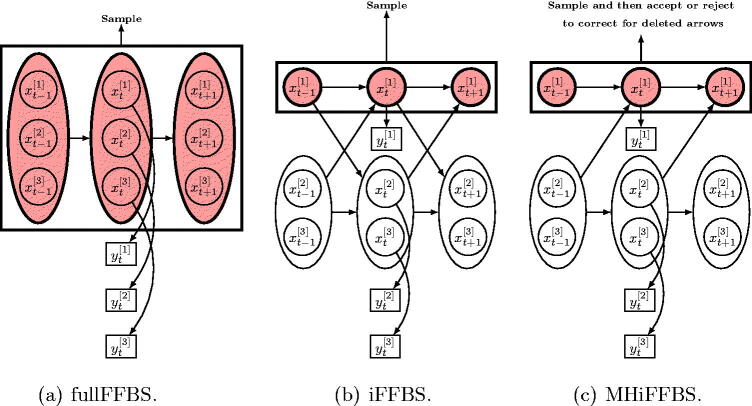
Strategies for simulating the hidden states in a coupled hidden Markov model: (a) standard FFBS algorithm where sampling is done for all chains jointly, (b) proposed iFFBS algorithm where the hidden states are sampled individually per chain conditionally on the rest, and (c) proposed MHiFFBS algorithm where sampling is also done individually per chain conditionally on the hidden states of the remaining chains; however, a MH acceptance step is introduced to correct for the fact that we deleted some between-chain arrows.

The most popular approach to exact Monte Carlo inference can be achieved by converting the CHMM into an equivalent HMM with $N^{C}$ states, where $X_{t}^{\left[1:C\right]}=\left(X_{t}^{\left[1\right]},X_{t}^{\left[2\right]},\ldots ,X_{t}^{\left[C\right]}\right)\in \Omega^{C}={\left\{s_{1},s_{2},\ldots ,s_{N}\right\}}^{C}$ denotes the state of the model at time *t*, as shown in Figure 2(a). In this case, the whole hidden state process can be updated from its full conditional, denoted by $\pi \left(X_{1:T}^{\left[1:C\right]}|Y_{1:T}^{\left[1:C\right]},\theta \right)$, in a single block by applying the standard FFBS algorithm (Carter and Kohn 1994; Chib 1996). This algorithm is based upon a forward recursion which calculates the filtered probabilities $P\left(X_{t}^{\left[1:C\right]}|Y_{1:t}^{\left[1:C\right]},\theta \right)$ for t=1,2,…,T. This is followed by a backward simulation step that first generates $X_{T}^{\left[1:C\right]}$ from $P\left(X_{T}^{\left[1:C\right]}|Y_{1:T}^{\left[1:C\right]},\theta \right)$ and then simulates the remaining $X_{t}^{\left[1:C\right]}$’s by progressing backward, simulating in turn $X_{t}^{\left[1:C\right]}$ from $P\left(X_{t}^{\left[1:C\right]}|X_{t+1}^{\left[1:C\right]},Y_{1:t}^{\left[1:C\right]},\theta \right)$, for t=T−1,T−2,…,1. We refer to this method as the fullFFBS.

The computational complexity of the fullFFBS algorithm is of the order $O(TN^{2C})$. Thus, particularly for a reasonably large number of chains or possible states, this method will be computationally demanding. As a result, several alternative methods have been proposed to solve the problem. The simplest approach to update the hidden states is to draw each one of the *C* × *T* state variables from its full conditional distribution. Such approach is referred to as single-site updates (see, e.g., (Dong, Pentland, and Heller 2012)). Thus we need to calculate *C* × *T* variables and each one requires O(N) time to compute giving an overall complexity of O(TNC). Despite being easy to implement, it has been shown by Scott (2002) that the single-site update algorithm can lead to extremely slow mixing in the resulting MCMC chains, due the high temporal dependence in the hidden state process.

An alternative method developed specifically for epidemic models was proposed by Spencer et al. (2015), which changes blocks of state components within a single chain, based on their current values. This method is a modification of O’Neill and Roberts (1999) and Gibson and Renshaw (1998), applied to discrete time models, and builds on the fact that individuals (represented by a single chain) remain in the same epidemic state for long periods. Briefly, for each chain successively one block of states **r** is chosen, and then one of three possible changes is proposed: Add, Remove, or Move. In an “Add” step, a period during which the individual does not change their state is identified and a subset of this period is proposed to have an alternative status. Likewise a “Remove” step proposes an alternative state for an entire episode in which the status is unchanged, joining two neighboring periods. A “Move” step moves an endpoint of such a block. Each of these changes proposes a new vector $r^{*}$, and the change is accepted with the usual MH acceptance probability.

The efficiency of the algorithm depends on the size of the blocks that are proposed to be updated. The main advantage of this method is that the computational requirement is very small since most of the hidden states are not updated. However, the downside is that this results in very slow mixing and requires many iterations to obtain sufficiently independent samples.

Assuming a sparse transition matrix is one way to speed up the FFBS algorithm, and such a method was proposed by Sherlock et al. (2013), where inference for each individual chain is performed conditioning on the hidden state vectors in all other chains. In this work, the authors imposed a structure on each chain’s transition matrix with transition probabilities depending on covariates through logistic regression. These covariates include the states of the other chains and other external factors. The approach presented here is similar to the one in Sherlock et al. (2013), however, their work requires the structure of transition matrices to be estimated or known in advance. In contrast, our approach explicitly takes into account the interaction between chains without imposing any structure on the transition matrix.

### 3.3 Proposed Methods

#### 3.3.1 Individual FFBS Gibbs Sampler

We propose a novel extension of the FFBS algorithm, where the hidden states are sampled individually per chain conditionally on the hidden states of the remaining chains, as opposed to the standard FFBS algorithm where sampling is done for all chains jointly. Under the conditional independence assumptions of our model, the full conditional distribution of $X_{1:T}^{\left[c\right]}$, for each c=1,2,…,C, can be factorized as

$$\begin{array}{c} P\left(X_{1:T}^{\left[c\right]}|X_{1:T}^{\left[-c\right]},Y_{1:T}^{\left[1:C\right]},\theta \right)=P\left(X_{T}^{\left[c\right]}|X_{1:T}^{\left[-c\right]},Y_{1:T}^{\left[1:C\right]},\theta \right)\\ \;\times \prod_{t=1}^{T-1}P\left(X_{t}^{\left[c\right]}|X_{t+1}^{\left[c\right]},X_{1:t+1}^{\left[-c\right]},Y_{1:t}^{\left[c\right]},\theta \right), \end{array}$$

where Bayes Theorem implies

(4) $$\begin{array}{c} P\left(X_{t}^{\left[c\right]}=x_{t}^{\left[c\right]}|X_{t+1}^{\left[c\right]}=x_{t+1}^{\left[c\right]},X_{1:t+1}^{\left[-c\right]},Y_{1:t}^{\left[c\right]},\theta \right)\\ \propto P\left(X_{t+1}^{\left[c\right]}=x_{t+1}^{\left[c\right]}|X_{t}^{\left[c\right]}=x_{t}^{\left[c\right]},X_{t}^{\left[-c\right]},\theta \right)\\ \times P\left(X_{t}^{\left[c\right]}=x_{t}^{\left[c\right]}|X_{1:t+1}^{\left[-c\right]},Y_{1:t}^{\left[c\right]},\theta \right), \end{array}$$

since the states of all chains at time *t* + 1 depend only on states at time *t*.

The rest of the calculation is concerned with determining the second mass function in Equation (4), which can be determined recursively for all *t* starting with *t* = 1. We refer to this term as the modified conditional filtered probability. The forward recursion is initialized at *t* = 1 with

(5) $$\begin{array}{c} P\left(X_{1}^{\left[c\right]}=x_{1}^{\left[c\right]}|X_{1:2}^{\left[-c\right]},Y_{1}^{\left[c\right]},\theta \right)\propto P\left(X_{1}^{\left[c\right]}=x_{1}^{\left[c\right]}|\theta \right)f_{x_{1}^{\left[c\right]}}\left(y_{1}^{\left[c\right]}|\theta \right)\\ \times \underset{\overset{\text{Transition}\text{probabilities}\text{of}\text{the}}{\text{remaining}\text{chains}\text{at}\text{time}t=2}}{\underset{︸}{\left[\prod_{c\prime \neq c}P\left(X_{2}^{\left[c\prime \right]}=x_{2}^{\left[c\prime \right]}|X_{1}^{\left[c\prime \right]}=x_{1}^{\left[c\prime \right]},X_{1}^{\left[-c\prime \right]},\theta \right)\right]}}. \end{array}$$

Since Ω is finite, the normalizing constant is given by the sum of the terms in the right-hand side of Equation (5). Then, for t=2,3,…,T−1, we repeat the following two steps:

Step 1. Compute the one-step ahead modified conditional predictive probabilities

(6) $$\begin{array}{c} P\left(X_{t}^{\left[c\right]}=x_{t}^{\left[c\right]}|X_{1:t}^{\left[-c\right]},Y_{1:t-1}^{\left[c\right]},\theta \right)\\ =\sum_{i\;\in \;\Omega }P\left(X_{t}^{\left[c\right]}=x_{t}^{\left[c\right]}|X_{t-1}^{\left[c\right]}=i,X_{t-1}^{\left[-c\right]},\theta \right)\\ \;\times P\left(X_{t-1}^{\left[c\right]}=i|X_{1:t}^{\left[-c\right]},Y_{1:t-1}^{\left[c\right]},\theta \right). \end{array}$$

Step 2. Compute the modified conditional filtered probabilities

(7) $$\begin{array}{c} P\left(X_{t}^{\left[c\right]}=x_{t}^{\left[c\right]}|X_{1:t+1}^{\left[-c\right]}=x_{1:t+1}^{\left[-c\right]},Y_{1:t}^{\left[c\right]},\theta \right)\\ \propto P\left(X_{t}^{\left[c\right]}=x_{t}^{\left[c\right]}|X_{1:t}^{\left[-c\right]},Y_{1:t-1}^{\left[c\right]},\theta \right)f_{x_{t}^{\left[c\right]}}\left(y_{t}^{\left[c\right]}|\theta \right)\\ \times \underset{\overset{\text{Transition}\text{probabilities}\text{of}\text{the}}{\text{remaining}\text{chains}\text{at}\text{time}t+1}}{\underset{︸}{\left[\prod_{c\prime \neq c}P\left(X_{t+1}^{\left[c\prime \right]}=x_{t+1}^{\left[c\prime \right]}|X_{t}^{\left[c\prime \right]}=x_{t}^{\left[c\prime \right]},X_{t}^{\left[-c\prime \right]}=x_{t}^{\left[-c\prime \right]},\theta \right)\right]}}, \end{array}$$

where computing the normalizing constant $\pi (Y_{t}^{\left[c\right]},X_{t+1}^{\left[-c\right]}|X_{1:t}^{\left[-c\right]},Y_{1:t-1}^{\left[c\right]},\theta )$ requires us to sum the right hand side of Equation (7) over the *N* possible values of $X_{t}^{\left[c\right]}$. Note that the last term in Equation (7) is calculated given $X_{t}^{\left[c\right]}$ and occurs due to $X_{t}^{\left[c\right]}$ connecting to $X_{t+1}^{\left[c\prime \right]}$ in the graph of Figure 2(b), for c′≠c.

The forward recursion is terminated at *t* = *T* with

(8) $$\begin{array}{c} P\left(X_{T}^{\left[c\right]}=x_{T}^{\left[c\right]}|X_{1:T}^{\left[-c\right]},Y_{1:T}^{\left[c\right]},\theta \right)\\ =\frac{P\left(X_{T}^{\left[c\right]}=x_{T}^{\left[c\right]}|X_{1:T}^{\left[-c\right]},Y_{1:T-1}^{\left[c\right]},\theta \right)f_{x_{T}^{\left[c\right]}}\left(y_{T}^{\left[c\right]}|\theta \right)}{\sum_{i\;\in \;\Omega }P\left(X_{T}^{\left[c\right]}=i|X_{1:T}^{\left[-c\right]},Y_{1:T-1}^{\left[c\right]},\theta \right)f_{i}\left(y_{T}^{\left[c\right]}|\theta \right)}. \end{array}$$

Once the filtered probabilities have been calculated and stored in a forward sweep, the hidden states for a given chain *c* can be simulated in a backward sweep, starting with $X_{T}^{\left[c\right]}$ from the modified filtered probability in Equation (8). Then for t=T−1,T−2,…,1 we iteratively sample a value for $X_{t}^{\left[c\right]}$ given our simulated value for $X_{t+1}^{\left[c\right]}$, from

$$\begin{array}{c} P\left(X_{t}^{\left[c\right]}=x_{t}^{\left[c\right]}|X_{t+1}^{\left[c\right]}=x_{t+1}^{\left[c\right]},X_{1:t+1}^{\left[-c\right]},Y_{1:t}^{\left[c\right]},\theta \right)\\ =\frac{P\left(X_{t+1}^{\left[c\right]}=x_{t+1}^{\left[c\right]}|X_{t}^{\left[c\right]}=x_{t}^{\left[c\right]},X_{t}^{\left[-c\right]},\theta \right)P\left(X_{t}^{\left[c\right]}=x_{t}^{\left[c\right]}|X_{1:t+1}^{\left[-c\right]},Y_{1:t}^{\left[c\right]},\theta \right)}{\sum_{i\;\in \;\Omega }P\left(X_{t+1}^{\left[c\right]}=x_{t+1}^{\left[c\right]}|X_{t}^{\left[c\right]}=i,X_{t}^{\left[-c\right]},\theta \right)P\left(X_{t}^{\left[c\right]}=i|X_{1:t+1}^{\left[-c\right]},Y_{1:t}^{\left[c\right]},\theta \right)}. \end{array}$$

This forward–backward procedure provides the full conditional distribution of the hidden Markov chain *c*, denoted by $P\left(X_{1:T}^{\left[c\right]}|Y_{1:T}^{\left[c\right]},X_{1:T}^{\left[-c\right]},\theta \right)$, in closed form. Therefore, we can use a Gibbs sampler where each chain is updated conditional on the current values of the remaining chains, the model parameters and the observed data. The algorithm is presented in Algorithm 1 and Figure 2(b) illustrates our proposed method, termed as individual FFBS (iFFBS) when the hidden states of chain *c* are updated.

In general, the scalability of the iFFBS algorithm is dictated by Equations (6) and (7). In Equation (6), a sum of *N* terms is calculated *N* times for each timepoint and individual, giving a scaling of $O(CN^{2}T)$. In Equation (7), a product with *C* – 1 terms is evaluated *N* times. Once all *C* individuals have been updated within the MCMC, this equation becomes quadratic in *C* to evaluate and so the iFFBS algorithm scales like $O(C^{2}N^{2}T)$. However, in most epidemic examples the product in Equation (7) can be rewritten as product over the *N*^2^ transition probabilities (e.g., probability of infection, recovery, etc.), raised to the power of the number of times the transition occurs. This is evaluated for each of the *N* possibilities for $x_{t}^{\left[c\right]}$. For such models, that is, models with joint transition probabilities that can be written as functions of sufficient statistics (which can be calculated initially and updated in O(1) time as each individual is updated), the iFFBS algorithm becomes linear in the number of individuals *C*. In this case the iFFBS algorithm scales as $O(CN^{3}T)$.

Algorithm 1: MCMC algorithm for the Markov model with iFFBS method.

1 Initialize: Draw θ∼π(θ) and generate

$$X_{1:T}^{\left[1:C\right]}∼P\left(X_{1:T}^{\left[1:C\right]}|\theta \right);$$

**2 for** *j = 1, 2, …, J* **do**

**3**  **for** *c = 1, 2, …, C* **do**

4   Draw $X_{1:T}^{\left[c\right]}∼\pi \left(X_{1:T}^{\left[c\right]}|Y_{1:T}^{\left[c\right]},X_{1:T}^{\left[-c\right]},\theta \right)$ with iFFBS;

**5**  **end**

6  Perform suitable MCMC update to sample

$$\theta ∼\pi \left(\theta |Y_{1:T}^{\left[1:C\right]},X_{1:T}^{\left[1:C\right]}\right);$$

**7 end**

#### 3.3.2 iFFBS Metropolis–Hastings Sampler

An important difference between the FFBS and iFFBS methods is that evaluating the filtered probabilities of chain *c* at time *t* < *T* for iFFBS involves the calculation of the transition probabilities of the remaining chains calculated at the next time point. Note that if these extra terms are omitted, then the iFFBS reduces to the standard FFBS applied to a single chain. This latter approximation was used by Sherlock et al. (2013) for modelling interactions of different diseases and by Fintzi et al. (2017) as part of an algorithm for updating the infection status of individuals in a continuous time epidemic model. We call this method the uncorrected-iFFBS because such an approximation can be made exact by introducing an extra MH step to correct for the fact that the hidden states are not sampled from their full conditionals. Note that failing to include the MH step may lead to poor behavior of the resulting MCMC chains; an example is presented in Supplementary Section A.

Motivated by epidemic examples, where the within chain dependence is much stronger than the between chain dependence, we propose using the uncorrected-FFBS applied to a single chain as a proposal distribution within a MH algorithm. More precisely we assume in Equation (7) that for all c′≠c, $P(X_{t+1}^{\left[c\prime \right]}|X_{t}^{\left[1:C\right]},\theta )\approx P(X_{t+1}^{\left[c\prime \right]}|X_{t}^{\left[-c\right]},\theta )$. This assumption implies the Bayesian network shown in Figure 2(c). Given the assumption of independence, the product terms in Equations (5) and (7) cancel out, and so the modified conditional filtered probabilities in the proposal distribution reduce to

$$\begin{array}{c} Q\left(X_{1}^{\left[c\right]}=x_{1}^{\left[c\right]}|X_{1:2}^{\left[-c\right]},Y_{1}^{\left[c\right]},\theta \right)\\ \;=\frac{P\left(X_{1}^{\left[c\right]}=x_{1}^{\left[c\right]}|\theta \right)f_{x_{1}^{\left[c\right]}}\left(y_{1}^{\left[c\right]}|\theta \right)}{\sum_{i\;\in \;\Omega }P\left(X_{1}^{\left[c\right]}=i|\theta \right)f_{i}\left(y_{1}^{\left[c\right]}|\theta \right)}, \end{array}$$

for the initial state and

$$\begin{array}{c} Q\left(X_{t}^{\left[c\right]}=x_{t}^{\left[c\right]}|X_{1:t+1}^{\left[-c\right]},Y_{1:t}^{\left[c\right]},\theta \right)\\ \;=\frac{P\left(X_{t}^{\left[c\right]}=x_{t}^{\left[c\right]}|X_{1:t}^{\left[-c\right]},Y_{1:t-1}^{\left[c\right]},\theta \right)f_{x_{t}^{\left[c\right]}}\left(y_{t}^{\left[c\right]}|\theta \right)}{\sum_{i\;\in \;\Omega }P\left(X_{t}^{\left[c\right]}=i|X_{1:t}^{\left[-c\right]},Y_{1:t-1}^{\left[c\right]},\theta \right)f_{i}\left(y_{t}^{\left[c\right]}|\theta \right)}. \end{array}$$

However, since we overlooked some between-chain dependencies our proposal Q is an approximation of the true full conditional. Therefore, we need to correct for the error of the approximation with a MH acceptance step. The detailed algorithm can be found in Algorithm 2. We refer to this proposed algorithm as MHiFFBS.

Algorithm 2: MCMC algorithm for the Markov model with MHiFFBS method.

1 Initialize: Draw θ∼π(θ) and generate $X_{1:T}^{\left[1:C\right]}∼P\left(X_{1:T}^{\left[1:C\right]}|\theta \right)$;

**2 for** *j = 1, 2, …, J* **do**

**3**  **for** *c = 1, 2, …, C* **do**

4   Propose $X_{1:T}^{[c]\;*}∼Q\left(\cdot |Y_{1:T}^{\left[c\right]},X_{1:T}^{\left[-c\right]},\theta \right)$;

5   Compute $a=\min(1,\frac{Q\left(X_{1:T}^{\left[c\right]}|Y_{1:T}^{\left[c\right]},X_{1:T}^{\left[-c\right]},\theta \right)}{Q\left(X_{1:T}^{[c]\;*}|Y_{1:T}^{\left[c\right]},X_{1:T}^{\left[-c\right]},\theta \right)}$ $\times \frac{\pi \left(X_{1:T}^{[c]\;*},X_{1:T}^{\left[-c\right]},\theta |Y_{1:T}^{\left[1:C\right]}\right)}{\pi \left(X_{1:T}^{\left[c\right]},X_{1:T}^{\left[-c\right]},\theta |Y_{1:T}^{\left[1:C\right]}\right)})$;

6   Draw u∼Uniform(0,1);

**7**   **if** u≤a **then**

8    Set $X_{1:T}^{\left[c\right]}=X_{1:T}^{[c]\;*}$;

**9**   **else**

10    Set $X_{1:T}^{\left[c\right]}=X_{1:T}^{\left[c\right]}$;

**11**   **end**

**12**  **end**

13  Perform suitable MCMC update to sample $\theta ∼\pi \left(\theta |Y_{1:T}^{\left[1:C\right]},X_{1:T}^{\left[1:C\right]}\right)$;

**14 end**

## 4 Analysis of Longitudinal Epidemic Data

### 4.1 Epidemic Model for *Escherichia coli* O157:H7

In this section, we demonstrate how CHMMs can be embedded within an individual-based epidemic model for the spread of infection among a population of individuals partitioned into groups. The example is based on a longitudinal study of *Escherichia coli (E. coli)* O157:H7 in cattle assigned into pens of the same size (Cobbold et al. 2007). We employ a discrete-time susceptible-infected-susceptible (SIS) model (Anderson and May 1991) for the spread of infection in a pen, where each individual in the population is assumed to belong to one of two states, susceptible or infected, for each day in the study.

More precisely, let $X_{t}^{\left[c,\;p\right]}\in \Omega =\{0,1\}$ denote the true infection status of individual c∈{1,2,…,C} in pen p∈{1,2,…,P} at day t∈T={1,2,…,T}, where $X_{t}^{\left[c,\;p\right]}=0$ represents the susceptible state, $X_{t}^{\left[c,\;p\right]}=1$ the infected state and *T* is the last day of the study. The transition probabilities for individual *c* in pen *p* are defined as

(9) $$\begin{array}{c} P\left(X_{t}^{\left[c,\;p\right]}=x_{t}^{\left[c,\;p\right]}|X_{t-1}^{\left[1:C,\;p\right]}=x_{t-1}^{\left[1:C,\;p\right]},\alpha ,\beta ,m\right)\\ =\{\begin{array}{cc} 1-\;\text{exp}\;\{-\alpha -\beta \sum_{c\prime =1}^{C}x_{t-1}^{\left[c\prime ,\;p\right]}\} & \text{if}x_{t-1}^{\left[c,\;p\right]}=0\text{and}x_{t}^{\left[c,\;p\right]}=1,\\ \;\text{exp}\;\{-\alpha -\beta \sum_{c\prime =1}^{C}x_{t-1}^{\left[c\prime ,\;p\right]}\} & \text{if}x_{t-1}^{\left[c,\;p\right]}=0\text{and}x_{t}^{\left[c,\;p\right]}=0,\\ \frac{m-1}{m} & \text{if}x_{t-1}^{\left[c,\;p\right]}=1\text{and}x_{t}^{\left[c,\;p\right]}=1,\\ \frac{1}{m} & \text{if}x_{t-1}^{\left[c,\;p\right]}=1\text{and}x_{t}^{\left[c,\;p\right]}=0, \end{array} \end{array}$$

for t=2,3,…,T. The parameter m≥1 denotes the mean infectious period and parameters α>0 and β>0 denote the external and within-pen infection rates, respectively, implying that pens are independent of one another. A generalization of the model that allows for transmission between pens is considered in Touloupou (2016). The first and last case in Equation (9) correspond to the infection (0↦1) and clearance (1↦0) probabilities, respectively. This parameterization defines a nonhomogeneous Markov model since it allows the probability of infection to depend on a sufficient statistic of the previous state of all individuals, namely the number of infected individuals. Finally, we assume that at the beginning of the study each animal is infected independently with probability $P(X_{1}^{\left[c,\;p\right]}=1|\nu )=\nu$.

The underlying infection process is not directly observed. Instead, for each individual we obtain the results of two diagnostic tests, taken at prespecified times. Let O⊆T denote the set of prescheduled observations times. Let $Y_{t}^{\left[c,\;p\right]}=\left(R_{t}^{\left[c,\;p\right]},F_{t}^{\left[c,\;p\right]}\right)$ be the observed results, possibly misclassified, of the diagnostic tests, $R_{t}^{\left[c,\;p\right]}$ for recto-anal mucosal swab (RAMS) and $F_{t}^{\left[c,\;p\right]}$ for fecal sample, where 1 denotes a positive and 0 a negative test result. Following Spencer et al. (2015), we assume that the observed test results are conditionally independent Bernoulli variables, with the success probabilities $\theta_{R}X_{t}^{\left[c,p\right]}$ and $\theta_{F}X_{t}^{\left[c,p\right]}$ given an individual with infection status $X_{t}^{\left[c,p\right]}$. Here, $\theta_{R}=P\left(R_{t}^{\left[c,\;p\right]}=1|X_{t}^{\left[c,\;p\right]}=1\right)$ is the sensitivity of the RAMS test and *θ_F_* is the sensitivity of the fecal test. Both test specificities are assumed to be 100%.

In the remainder of this section, we perform a series of simulations to assess the efficiency of existing and proposed methods for updating the hidden infection states. Particular focus is given on how these methods are affected by dimensionality that is, when the total number of individuals in the population and the study period increase. In Section 4.2, we apply the methods to data simulated from the Markov model in Equation (9) with a Geometric distribution for the infection period (see also Supplementary Section A). In Section 4.3, we relax the Markovian assumption by allowing the duration to have a negative binomial distribution. This leads to a semi-Markov model in which the duration of infection depends on how long an individual has been infected. Finally, in Section 4.4 the performance of our methods is assessed on the real *E. coli* O157 dataset, considering both Markov and semi-Markov models. The simulations, analyses, and graphics rely upon the foreach (Microsoft Corporation and Weston 2017), doParallel (Microsoft Corporation and Weston 2018), ggplot2 (Wickham 2016), and tools available in the standard R distribution (R Core Team 2016).

### 4.2 Simulation Studies: Markov Model

The initial simulated dataset consists of observations from *P* = 20 pens, each containing *C* = 8 cattle and the study period is set to *T* = 99 days as in the real *E. coli* O157:H7 dataset (Cobbold et al. 2007). First, we generated the hidden infection states according to the model defined in Equation (9), with an external transmission rate α=0.009, within-pen transmission rate β=0.01, mean infectious period *m* = 9 days, and initial probability of infection ν=0.1. We then generate RAMS and fecal tests from the population according to the actual sampling frame employed in the real dataset; sampling on average twice per week. Finally, the RAMS and fecal test sensitivities are assumed to be $\theta_{R}=0.8$ and $\theta_{F}=0.5$, respectively. These parameter values are motivated by the results obtained by Spencer et al. (2015) who previously analyzed the same data.

We then estimated the parameters in the Markov model using the following vague prior distributions: α,β∼ Ga(1,1), m−1∼ Ga(0.01, 0.01), and $\nu ,\theta_{R},\theta_{F}∼$ Beta(1,1). We drew samples from the joint posterior of the hidden states and model parameters with the MCMC scheme described in Section 3.1, using each method for updating the hidden states. The model parameters *ν*, *θ_R_*, and *θ_F_* were updated using Gibbs steps and the remaining parameters were updated jointly using Hamiltonian Monte Carlo (HMC; Neal 2011), for details see Supplementary Section B. For each method, we ran the algorithm for 11,000 iterations, removing the first 1000 as a burn-in. Each procedure was repeated 200 times to provide an empirical Monte Carlo estimate of the variation in each approach.

Figure C.1 in Supplementary Section C shows the estimated total number of infected individuals over time, along with 95% credible intervals, as obtained from the 200 runs. The five methods provide almost identical results and all of them contain the true total number of infected individuals within the credible intervals. Therefore, a comparison of the different approaches can be based on the mixing properties and the required computational effort of each. Mixing can be measured in terms of autocorrelation of the Markov chains whereas the computational effort is given by the total time required for one iteration of the MCMC. In the following results we chose our summary statistic to be the total infection pressure $\text{TIP}=\sum_{p=1}^{P}\sum_{c=1}^{C}\sum_{t=1}^{T}x_{t}^{\left[c,\;p\right]}$, in order capture the information over all *T* periods of the study.

**Fig. 3:**
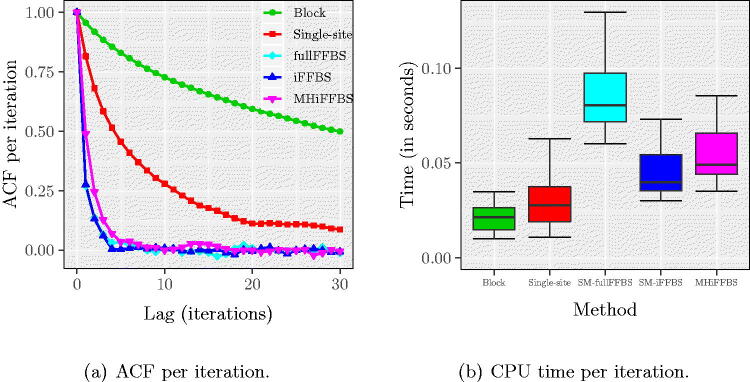
(a) Autocorrelation function of TIP and (b) CPU time per iteration for the Markov epidemic model. ACF plots in the left panel are the average across 200 replicates. Quantiles in the right panel are obtained from the same 200 runs. These plots show that the fullFFBS, iFFBS, and MHiFFBS methods have very good mixing properties but are more computationally expensive than the remaining methods.

In Figure 3(a), we see the autocorrelation function (ACF) for TIP, averaged across the 200 different runs in each method. We see that the fullFFBS, iFFBS, and MHiFFBS methods have very good mixing properties since the autocorrelation function drops rapidly. In contrast, the block proposals and single-site updates produced highly correlated samples with the ACF being greater than zero even after 30 iterations of the MCMC. For the block proposals, slow mixing was due to only a few states being updated at each iteration of the MCMC; for the single-site method slow mixing was caused by the strong correlation between hidden states. However, the block proposal method was the fastest, as can be seen in Figure 3(b). The computationally most demanding method was fullFFBS due to the summation over all of the 2^8^ possible states.

**Fig. 4:**
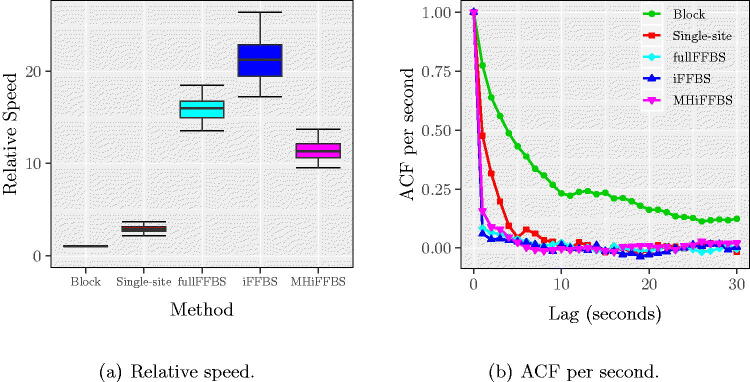
(a) Relative speed and (b) ACF per second for TIP for the Markov epidemic model, based on 200 replications. We observe that the iFFBS method outperforms the remaining methods when we consider the relative speed as a measure of performance.

Computation efficiency is a combination of mixing and computation time. We use the relative speed which is defined as follows. First, for each method we calculate the time normalized effective sample size (tESS), taken as the ratio of effective sample size (ESS) from 10,000 MCMC iterations and the CPU time required per iteration. Then, we divide the tESS of each method with the worst observed tESS to obtain the relative speed. Hence, the relative speed has a minimum value of 1 which corresponds to the computationally least efficient method, and any number bigger than 1 reflects the gains using a particular method compared to the worst. In Figure 4(a), we show the relative speed of each method as obtained from the 200 different runs. We observe that among competing methods, the iFFBS method best combines the desired properties of mixing and computational speed, followed by the fullFFBS and the MHiFFBS methods. Using block proposals was the least efficient method as it had the smallest relative speed in all 200 replicates. This finding is confirmed in Figure 4(b) where we show the ACF per second.

**Fig. 5:**
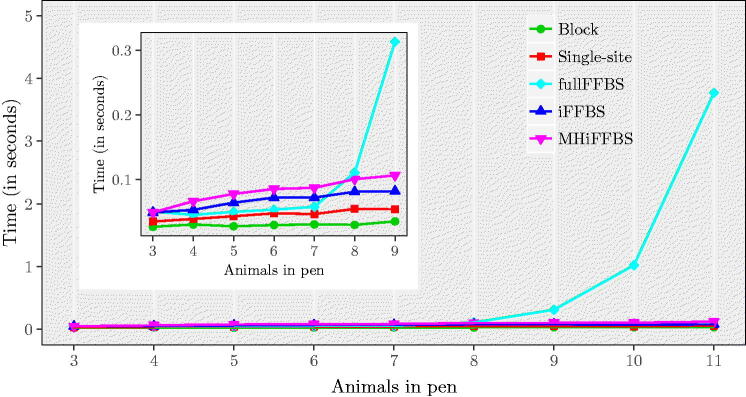
CPU time per iteration as a function of the total number of cattle per pen *C*, for the Markov epidemic model. The subpanel provides an enlargement for 3–9 animals in pen, illustrating more clearly that the fullFFBS algorithm scales poorly.

In the next set of simulations, we study how computation time is affected as we vary the total population size by increasing the cattle size per pen. We use our initial simulation settings and generate one dataset for different numbers of Markov chains, C=3,4,…,11. Figure 5 illustrates the time taken per iteration of the five different methods as the number of animals in a pen varies. We see that for the fullFFBS the computational time grows exponentially with *C*. The other methods are only affected linearly when *C* increases. As before, we assess computational efficiency with the relative speed. Results are summarized in Table C.1(a) in Supplementary Section C. Note that despite being the computationally most efficient for small C, the performance of fullFFBS drops with *C* and eventually for *C* = 11 it has the lowest relative speed. For *C* > 6, the iFFBS method outperforms the remaining methods. To study the influence of the study length on the performance of each method, we repeat our simulation study for different values of *T*. Results are given in Table C.1(b). Again, the iFFBS method is the one that scores higher in terms of relative speed, followed in order by fullFFBS, MHiFFBS, single-site, and block proposals.

**Fig. 6:**
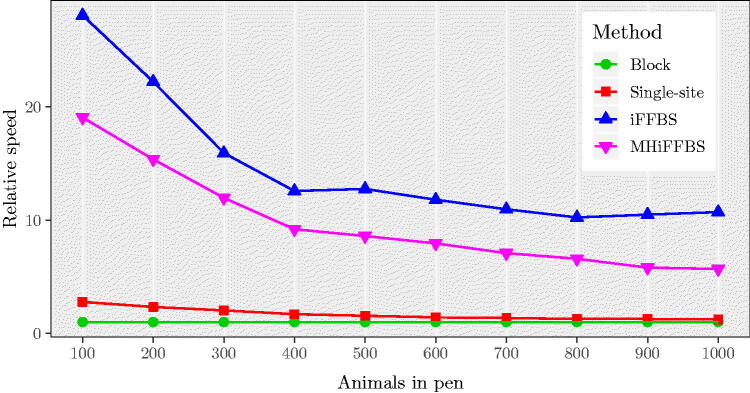
Median relative speed comparison of four methods in the Markov model for large datasets with values for C=100,200,…,1000, based on 200 simulations. As observed before, iFFBS outperforms the other methods considered.

In our simulations so far we have evaluated the performance of the five methods for data of moderate dimensionality; however, many applications involve datasets with substantially more individuals. Application of the fullFFBS method quickly becomes computationally prohibitive and cannot be included. Figure 6 considers simulations with population sizes between 100 and 1000. As before, the iFFBS outperforms the other methods whereas the least efficient is the block update method with a relative speed equal to 1 in all scenarios. The gains of using the iFFBS algorithm are higher in the first scenario with 100 animals per pen, where the method has a relative speed of 28.09. However, the differences in the computational efficiency among methods become less profound as the total number of individuals per pen increases. For example, in the last scenario (*C* = 1000) the relative speed of iFFBS algorithm drops to 10.71. Finally, we investigate the performance of the MHiFFBS method subject to varying the number of individuals in pen. The results are summarized in Figure C.2 in Supplementary Section C, where we report the variability in the median acceptance rate (over all individuals) as obtained from the 200 replications. We observe a small decline in the acceptance rate as the total number of individuals increases. In particular, we see that the rates are bigger than 0.84 for all values of *C* considered.

### 4.3 Simulation Studies: Semi-Markov Model

In a departure from the previous Markov model, we assume that the time an individual remains infected has a two-parameter Negative Binomial distribution and hence

$$\begin{array}{c} P\left(X_{\left(t+1):(t+s\right)}^{\left[c,\;p\right]}=1_{s},X_{t+s+1}^{\left[c,\;p\right]}=0|X_{t}^{\left[c,\;p\right]}=0,X_{t+1}^{\left[c,\;p\right]}=1\right)\\ \;={\left(\frac{\kappa }{\kappa +m-1}\right)}^{\kappa }\frac{\Gamma (\kappa +s-1)}{\left(s-1)!\Gamma (\kappa \right)}{\left(\frac{m-1}{\kappa +m-1}\right)}^{s-1}, \end{array}$$

where κ>0 is the shape parameter, m≥1 is the mean duration of infection and $1_{s}$ is a vector of *s* ones. In this semi-Markov model the time remaining until recovery depends on how long an individual has been infected. The infection probability remains unchanged.

Bayesian inference for the semi-Markov model can proceed as follows. Regarding the update of the hidden states, the block proposals and the single-site methods can be applied without any modification. For the fullFFBS and iFFBS methods the necessary Markov property is not valid, and the two algorithms cannot be applied directly. Therefore, we extend the methodology for updating the hidden states by considering an independence sampler within the MCMC algorithm. Our approach takes advantage of the availability of the full conditionals in the CHMM, by using them as a proposal in the update. More specifically, proposals are made assuming *κ* = 1, corresponding to the Geometric distribution as considered before, and introducing an MH acceptance step to correct for the discrepancy. The efficiency of the algorithm therefore depends on how close the real value of *κ* is to 1. The extended algorithms for fullFFBS (called SM-fullFFBS) and iFFBS (called SM-iFFBS) are shown in Algorithms D.1 and D.2, respectively and are further detailed in Supplementary Section D.1. The MHiFFBS method can also be applied with proposals using *κ* = 1 and since it already includes a MH step, no further corrections are needed. The mixing of these algorithms for semi-Markov models may be improved through the introduction of auxiliary states, for example, via Erlang’s method of stages (Barbour 1976) or, more generally, phase-type distributions (Neuts 1975), but at the price of greater computation time. For example, if *κ* = 4 the negative binomial distribution can be represented as a sum of 4 independent and identically distributed geometric distributions, each represented by a state, and the iFFBS algorithm provides a Gibbs step. However, since the computational time of iFFBS is quadratic in the number of states the best relative speed may be obtained by using the SM-iFFBS with a smaller number of states.

**Fig. 7:**
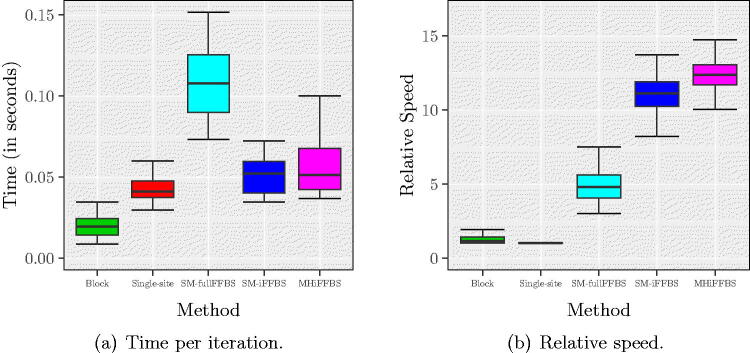
(a) CPU time per iteration and (b) relative speed for the semi-Markov epidemic model. Quantiles in both left and right panels are obtained from 200 different replicates. In these scenarios, the least efficient method is the one based on single-site updates.

In this section, we repeat the simulation analyses of Section 4.2 assuming the semi-Markov model. The shape parameter *κ* is set to 1.6 as estimated from the real data by Spencer et al. (2015). We used a Ga(0.01,0.01) prior for *κ* and estimate it alongside the remaining parameters in the MCMC. In particular α,β,m, and *κ* are updated jointly with HMC; see Supplementary Section D.2 for details. As before, we found little difference in the estimated number of infected individuals across the methods and these estimates were again close to the real values (Figure E.1 in Supplementary Section E). Figure 7 compares CPU timings and relative speeds. In this semi-Markov model, both block updates and MHiFFBS methods could be applied without modification and therefore required approximately the same time per iteration; the remaining methods were slowed down due to the modifications explained above (see Figure 7(a)). In terms of relative speed, MHiFFBS had a slightly higher median compared to SM-iFFBS which was second best, followed by SM-fullFFBS, block proposals, and single-site methods, Figure 7(b). However, the best two had overlapping credible intervals depending on how important the missing arrows were; if the arrows were very important then it is better to use SM-iFFBS and if unimportant then MHiFFBS may be best. Furthermore, the single-site method appeared to be the least efficient because in the semi-Markov model the history of each individual must be represented explicitly in the full conditionals leading to a significant increase in computational effort. Comparing Figure 7(b) with Figure 4(a) we conclude that the gains of using the proposed algorithms drop when we move from the Markov to the more complex semi-Markov model. For SM-iFFBS this fact is due to the extra MH step introduced within the sampler.

Results of relative speed for several values of *C* and *T* are shown in Table E.1(a) and (b), respectively, in Supplementary Section E. For the semi-Markov model the SM-iFFBS approach has similar performance to the MHiFFBS. MHiFFBS had the highest relative speed in 15 out of the 18 simulated datasets whereas SM-iFFBS was the most efficient in 2 out of 18 occasions; nevertheless the differences were small on most occasions. Another interesting observation is that the block update method now produces a better relative speed than the single-site method in 17 out of 18 simulations. For large datasets, we observe superiority of the two proposed methods in relative speed (Figure E.2 in Supplementary Section E), resulting in a different pattern compared to the Markov case (Figure 6). This difference occurs because the relative speed is compared to the slowest method and the single-site update requires considerably more computational effort in the semi-Markov model.

**Fig. 8:**
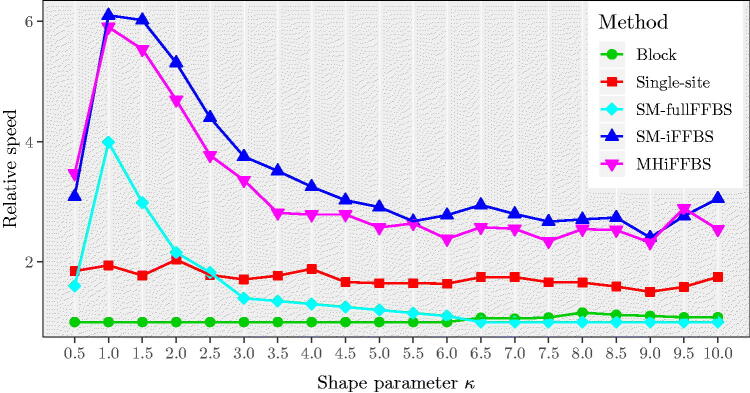
Median relative speed comparison of the five methods for different values of κ=0.5,1,1.5,…,10 for the semi-Markov model with 8 individuals. The relative speed curves for SM-fullFFBS, SM-iFFBS, and MHiFFBS are peaked at *κ* = 1.

Finally, we carried out a sensitivity analysis to assess the effect that the additional parameter *κ* has on the relative speed values, by simulating datasets with values for *κ* from 0.5 through to 10, increasing by 0.5 each time. For each value of *κ* we obtained an estimate of the relative speed, based on 200 simulated datasets. Results are shown in Figure 8. Comparing the five methods, we see that our two proposed novel methods outperform the remaining methods and that they give similar estimates of the relative speed for all scenarios considered. Moreover, for values of *κ* close to 1 the SM-iFFBS, SM-fullFFBS, and MHiFFBS provided much higher values of relative speed. Additionally, the performance of SM-fullFFBS drops as *κ* increases and eventually for κ>6 it was found to have the lowest relative speed. This poor performance is due to the fact that the SM-fullFFBS proposes all of the periods of infection simultaneously, and so deviations from the true infectious period distribution are noticeable and the acceptance rate is low, as we can see in Figure E.4 of the Supplementary Section E. However, the SM-iFFBS and MHiFFBS propose only a small number of infectious periods before each accept/reject step, and so deviations from the true infectious period distribution are not as important and the acceptance rate remains high, with a value of roughly 60% for both methods.

### 4.4 Summary of *E. coli* O157:H7 Data Analysis

In this section, we use the existing and proposed methods for the analysis of the real *E. coli* O157:H7 dataset presented in Section 4.1. We consider both Markov and semi-Markov models. A full description of the analysis summarized here can be found at Supplementary Section F. In terms of parameter estimation, the five methods provide almost identical estimates (Table F.1 in Supplementary Section F) and in close agreement with results presented by Spencer et al. (2015) who previously analyzed the same data. Overall, our analyses suggest that the proposed methods outperform the other methods in terms of computational efficiency as indicated by the median relative speed shown in Figure F.1 in Supplementary Section F. The same conclusions were reached in the simulation studies.

## 5 Discussion

In this article, we have considered the problem of Bayesian estimation of the hidden states in CHMMs, an extension of classical HMMs that allow for interactions between the hidden states of each chain. In particular, we have compared existing methods in a real application and introduced two new approaches, the iFFBS and MHiFFBS algorithms. We have extended the methods to a CHSMM in which the hidden process can remain in a given state for a non-memoryless duration. The computational efficiency was compared in the context of modelling the dynamics of an infectious disease using both a Markov and a semi-Markov model for the duration of infection.

In our simulation studies, we found the iFFBS algorithm outperformed the existing methods. It balances the desired properties of good mixing and low CPU time and thus proved to be computationally most efficient. This is achieved by exploiting the dependence structure in the model, where the within chain dependence is much stronger than the between chain dependence. The findings were stronger for the Markov model but also held in the semi-Markov case. Additionally, we have also demonstrated that the proposed iFFBS method can scale well for big datasets with order $CN^{3}T$ for epidemic models and at worst order $C^{2}N^{2}T$; as opposed to the standard FFBS algorithm which scales like $O(N^{2C}T)$ and cannot be applied when the number of chains in the CHMM is growing.

The importance of the proposed approaches is further demonstrated in Touloupou (2016), where we have illustrated how iFFBS can be used for inference in epidemic models with more complex dynamics, for example, a model allowing for interactions between neighboring pens; some additional terms appear in the full conditional distribution to account for interactions between animals in different pens. More specifically, the updates for a chain *c* are done conditionally not only on the chains of the remaining subjects in the pen but also conditionally on the chains of individuals in the neighboring pens. As a result, the modified filtered probabilities additionally include the transition probabilities of subjects in neighboring pens.

There are several ways in which the proposed methodologies can be extended. In the current approach, we update the states of a single chain given the rest. One alternative is to apply a block update scheme, where small subsets of chains are jointly sampled from their full conditionals. This approach would be particularly effective when there is some underlying structure between the chains that increases the dependence within the blocks, such as individuals grouped into households in an epidemic context. Furthermore, in this article we have limited our discussion on the deterministic Gibbs sampler, in which individual chains are sampled iteratively. However, the iFFBS algorithm unlocks the possibility of an adaptive random scan Gibbs sampler (Łatuszyński et al. 2013), that learns the individuals that need to be updated more frequently. Recent work by Chimisov, Łatuszyński, and Roberts (2018) develops such an approach and demonstrates substantial improvements in computational efficiency for a Markov switching model, which is similar in spirit to the CHMMs discussed here. For small epidemics within large populations, an adaptive iFFBS sampler for the missing data has the potential for immense improvements in computational efficiency, due to the fact that most individuals do not take part in the epidemic and therefore need their infection status updated only rarely.

## Supplementary Materials

**Supplement:** Additional plots/results for the simulations covered in the article, real data analysis and the underlying algorithms details for the implementation. (.pdf file)

**Simulations:** Code to replicate the simulations and to run the proposed algorithms described in the article. (.zip file)

## Supplementary Material

Supplemental Material
