# Supplementary material for: Scalable Bayesian Inference for Coupled Hidden Markov and Semi-Markov Models
Source: J Comput Graph Stat. 2019 Sep 18;29(2):238–49. doi: 10.1080/10618600.2019.1654880 (PMC7455056; doi:10.1080/10618600.2019.1654880)
Supplement: Supplemental Material [file UCGS_A_1654880_SM2590.zip › SupplementaryMaterial/Supplement.pdf]

Supplementary Material: Scalable Bayesian inference for coupled hidden Markov and semi-Markov models

Panayiota Touloupou, Bärbel Finkenstädt Rand, and Simon E. F. Spencer

Department of Statistics, University of Warwick

A Toy example: three interacting individuals

In this Section we provide an example of the poor mixing properties that may occur when we sample directly from the approximated full conditionals of the hidden individual disease states in the CHMM, without correcting with a MH acceptance step. We consider a dataset with a single pen and $C = 3$ individuals for sampling interval of $T = 11$ days and obtain imperfect test results at days $t \in O = \{1, 4, 8, 11\}$. The data are simulated from the Markov model described in Section 4.1 where we do not allow for transmission of the disease from any other source apart from within-pen transmission, achieved by setting the external infection rate $\alpha = 0$. The rest of the model parameters are set according to the posterior median of the real data analysis obtained by Spencer et al. (2015). We plot the observed data along with the true infection states in Figure A.1.

We investigate the efficiency of the methods described in Section 3 as well as the standard version of the FFBS (uncorrected-iFFBS) that does not account for approximation of the full conditionals, in the case that update of the hidden states is done individual-by-individual. We evaluate the mixing properties of the different methods by looking at the estimated posterior probability of infection for each individual per day over the entire sampling period. Results are also shown in Figure A.1. We see that all methods provide identical results except from the uncorrected-iFFBS method that converges to a different distribution. We further apply the MH correction to the uncorrected-iFFBS method, called

MHiFFBS as discussed in Section 3.3.2, and see that the method converges to the same values as the rest of the methods, with an acceptance rate of 0.5 for individual 1, 0.75 for individual 2 and 0.95 for individual 3.

The poor observed performance can be explained by the example shown in Figure A.2. In this figure we show the sampled hidden disease states at iterations j and $j+1$ of the MCMC using the uncorrected-iFFBS method. In the middle panel, we observe that even though at day 2 the disease has died out, it re-appears at day 3. However, this should be impossible based on the model assumption that does not allow for external transmission of the disease. The reason for this phenomenon is that the sampler ignores the carriage states of other individuals in the next day when it calculates the filtered probabilities. As an example, in the update of animal 1 at day 2 the sampler gives a value of 0; nevertheless, if it accounted for the infection state of animal 2 at day 3 then it should give a value of 1 to ensure that there is at least one individual who can transmit the disease to the next day. This is corrected at the update of individual 2 at day 2, which finds that the animal is infected the following day 3 and hence needs to be infected at day 2 as well (bottom panel).

B MCMC details for the Markov model

In this section we provide the details of the MCMC algorithm presented in Section 4.2, used to generate samples from the posterior of the coupled hidden Markov model. Simulation of the hidden states $\mathbf{X}_{1:T}^{[1:C]}$ has been already discussed in the main body. We now give details for the remaining model parameters.

B.1 Updating the transmission parameters

The full conditional distribution of the initial infection parameter ν, is:

$$\pi(\nu \mid \mathbf{Y}_{1:T}^{[1:C]}, \mathbf{X}_{1:T}^{[1:C]}, \tilde{\alpha}, \tilde{\beta}, \tilde{m}, \theta_R, \theta_F) \propto \nu^{b_\nu - 1}(1-\nu)^{c_\nu - 1} \prod_{p=1}^{P}\prod_{c=1}^{C}\left[\nu^{x_1^{[c,\,p]}}(1-\nu)^{1-x_1^{[c,\,p]}}\right]$$
$$= \nu^{\sum_{p=1}^{P}\sum_{c=1}^{C} x_1^{[c,\,p]} + b_\nu - 1}(1-\nu)^{\sum_{p=1}^{P}\sum_{c=1}^{C}\left(1-x_1^{[c,\,p]}\right)+c_\nu - 1}.$$

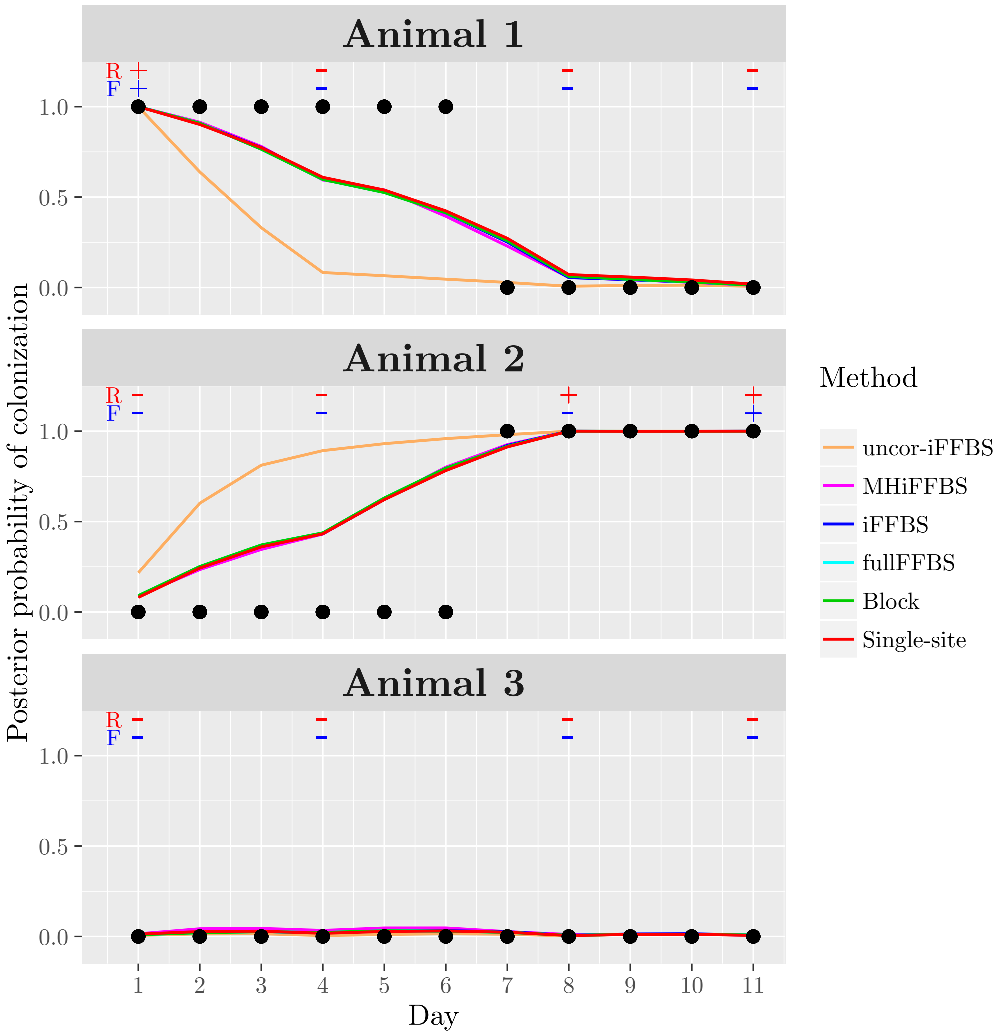

Figure A.1: Posterior probability of infection for individuals in the simulated dataset of Section A, over the entire sampling period of 11 days. Black dots represent the true infection states (1 for infected, 0 for susceptible). For reference we also show test results taken at days 1, 4, 8, 11.

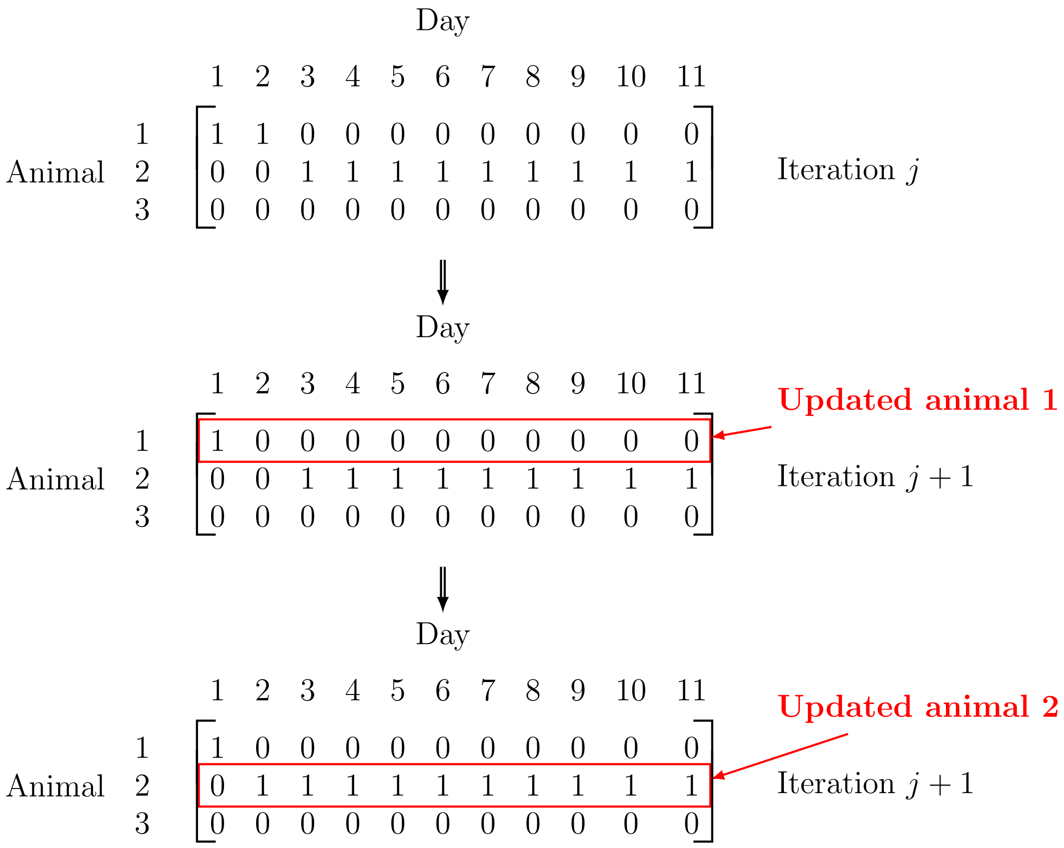

Figure A.2: Snapshots of the Gibbs hidden state updates with uncorrected-iFFBS method. Upper panel shows the states after iteration j of the MCMC is complete. Middle panel shows the hidden states after individual 1 has been updated at iteration $j+1$. Finally, the bottom panel represents the same information after the update of the second individual.

Hence we draw $\nu \mid \cdot \sim \text{Beta}\left(\sum_{p=1}^{P}\sum_{c=1}^{C} x_1^{[c,p]} + b_\nu, \sum_{p=1}^{P}\sum_{c=1}^{C}\left(1 - x_1^{[c,p]}\right) + c_\nu\right)$. For $\tilde{\alpha} = \log(\alpha)$, $\tilde{\beta} = \log(\beta)$ and $\tilde{m} = m - 1$ the joint full conditional is given up to a multiplicative constant as:

$$\pi(\tilde{\alpha}, \tilde{\beta}, \tilde{m} \mid \mathbf{Y}_{1:T}^{[1:C]}, \mathbf{X}_{1:T}^{[1:C]}, \nu, \theta_R, \theta_F)$$
$$\propto \prod_{p=1}^{P}\prod_{t=2}^{T}\left[\left(\exp\left\{-e^{\tilde{\alpha}} - e^{\tilde{\beta}}\sum_{c=1}^{C} x_{t-1}^{[c,p]}\right\}\right)^{N_{00}^{p}(t)}\left(\frac{\tilde{m}}{\tilde{m}+1}\right)^{N_{11}^{p}(t)}\left(\frac{1}{\tilde{m}+1}\right)^{N_{10}^{p}(t)}\right.$$
$$\left.\times\left(1 - \exp\left\{-e^{\tilde{\alpha}} - e^{\tilde{\beta}}\sum_{c=1}^{C} x_{t-1}^{[c,p]}\right\}\right)^{N_{01}^{p}(t)}\right] \times \pi_\alpha\big(e^{\tilde{\alpha}}\big) \times e^{\tilde{\alpha}} \times \pi_\beta\big(e^{\tilde{\beta}}\big) \times e^{\tilde{\beta}} \times \pi_{\tilde{m}}(\tilde{m}),$$

where $N_{kj}^{p}(t)$ the number of individuals in pen p who were in state k at time $t-1$ and in state j at time t, for $k, j \in \{0, 1\}$ and π_V is the prior distribution of parameter V.

This distribution cannot be solved analytically and therefore we use HMC to update these parameters. We have that the partial derivatives are given by:

$$\frac{\partial \log \pi(\tilde{\alpha}, \tilde{\beta}, \tilde{m} \mid \cdot)}{\partial \tilde{\alpha}} = \sum_{p=1}^{P}\sum_{t=2}^{T}\left[N_{01}^{p}(t) \times e^{\tilde{\alpha}} \times \frac{\exp\left\{-e^{\tilde{\alpha}} - e^{\tilde{\beta}}\sum_{c=1}^{C} x_{t-1}^{[c,p]}\right\}}{1 - \exp\left\{-e^{\tilde{\alpha}} - e^{\tilde{\beta}}\sum_{c=1}^{C} x_{t-1}^{[c,p]}\right\}}\right.$$
$$\left. - N_{00}^{p}(t) \times e^{\tilde{\alpha}}\right] + \frac{\partial \log \pi_\alpha(e^{\tilde{\alpha}})}{\partial \tilde{\alpha}} + 1,$$

$$\frac{\partial \log \pi(\tilde{\alpha}, \tilde{\beta}, \tilde{m} \mid \cdot)}{\partial \tilde{\beta}} = \sum_{p=1}^{P}\sum_{t=2}^{T}\left[N_{01}^{p}(t) \times e^{\tilde{\beta}} \times \sum_{c=1}^{C} x_{t-1}^{[c,p]} \times \frac{\exp\left\{-e^{\tilde{\alpha}} - e^{\tilde{\beta}}\sum_{c=1}^{C} x_{t-1}^{[c,p]}\right\}}{1 - \exp\left\{-e^{\tilde{\alpha}} - e^{\tilde{\beta}}\sum_{c=1}^{C} x_{t-1}^{[c,p]}\right\}}\right.$$
$$\left. - N_{00}^{p}(t) \times e^{\tilde{\beta}} \times \sum_{c=1}^{C} x_{t-1}^{[c,p]}\right] + \frac{\partial \log \pi_\beta(e^{\tilde{\beta}})}{\partial \tilde{\beta}} + 1,$$

$$\frac{\partial \log \pi(\tilde{\alpha}, \tilde{\beta}, \tilde{m} \mid \cdot)}{\partial \tilde{m}} = \sum_{p=1}^{P} \sum_{t=2}^{T} \left[\frac{N_{11}^{p}(t)}{\tilde{m}} + \frac{N_{11}^{p}(t) + N_{10}^{p}(t)}{\tilde{m}+1} \right] + \frac{\partial \log \pi_{\tilde{m}}(\tilde{m})}{\partial \tilde{m}}.$$

We use a fixed number of leapfrog steps $L = 30$ and adopt the stepsize ϵ during burn-in to obtain an acceptance rate of roughly 65% as suggested by Neal (2011).

B.2 Updating the observation parameters

The full conditional of θ_R is:

$$\begin{aligned} &\pi(\theta_R \mid \mathbf{Y}_{1:T}^{[1:C]}, \mathbf{X}_{1:T}^{[1:C]}, \tilde{\alpha}, \tilde{\beta}, \tilde{m}, \nu, \theta_F) \\ &\qquad \propto \theta_R^{b_{\theta_R}-1} (1-\theta_R)^{c_{\theta_R}-1} \prod_{p=1}^{P} \prod_{c=1}^{C} \prod_{\substack{x_t^{[c,\,p]}=1 \\ t \in O}} \left[\left(\theta_R\right)^{\mathrm{r}_t^{[c,\,p]}} \left(1-\theta_R\right)^{1-\mathrm{r}_t^{[c,\,p]}} \right], \end{aligned}$$

and for θ_F it is:

$$\begin{aligned} &\pi(\theta_F \mid \mathbf{Y}, \mathbf{X}, \tilde{\alpha}, \tilde{\beta}, \tilde{m}, \nu, \theta_R) \\ &\qquad \propto \theta_F^{b_{\theta_F}-1} (1-\theta_F)^{c_{\theta_F}-1} \prod_{p=1}^{P} \prod_{c=1}^{C} \prod_{\substack{x_t^{[c,\,p]}=1 \\ t \in O}} \left[\left(\theta_F\right)^{\mathrm{f}_t^{[c,\,p]}} \left(1-\theta_F\right)^{1-\mathrm{f}_t^{[c,\,p]}} \right]. \end{aligned}$$

Hence we draw θ_R and θ_F:

$$\begin{aligned} \theta_R \mid \cdot \;&\sim\; \text{Beta}\left(\sum_{p=1}^{P} \sum_{c=1}^{C} \sum_{\substack{x_t^{[c,\,p]}=1 \\ t \in O}} \mathrm{r}_t^{[c,\,p]} + b_{\theta_R}, \sum_{p=1}^{P} \sum_{c=1}^{C} \sum_{\substack{x_t^{[c,\,p]}=1 \\ t \in O}} \left(1-\mathrm{r}_t^{[c,\,p]}\right) + c_{\theta_R} \right), \\ \theta_F \mid \cdot \;&\sim\; \text{Beta}\left(\sum_{p=1}^{P} \sum_{c=1}^{C} \sum_{\substack{x_t^{[c,\,p]}=1 \\ t \in O}} \mathrm{f}_t^{[c,\,p]} + b_{\theta_F}, \sum_{p=1}^{P} \sum_{c=1}^{C} \sum_{\substack{x_t^{[c,\,p]}=1 \\ t \in O}} \left(1-\mathrm{f}_t^{[c,\,p]}\right) + c_{\theta_F} \right). \end{aligned}$$

C Additional results for the Markov model

In this section we provide additional results for the analysis of the simulated data from the Markov model of Section 4.2. The estimated number of infected individuals over time, along with 95% credible intervals, as obtained from one of the 200 runs for each of the five

methods considered is shown in Figure C.1. Results of relative speed for several values of C and T are shown in Table C.1(a) and Table C.1(b) respectively. Finally, the performance of the MHiFFBS method is assessed in Figure C.2 where we present the median acceptance rate for data generated with $C = 100, 200, \ldots, 1000$. Overall, we see a slight decrease in the acceptance rates with the number of individuals in pen.

D MCMC details for the semi-Markov model

In this section we provide the details of the MCMC algorithm, used to generate samples from the posterior of the semi-Markov transmission model presented in Section 4.3.

D.1 SM-fullFFBS and SM-iFFBS algorithms

In Algorithm D.1 and D.2 we present the detailed algorithm for the SM-fullFFBS and SM-iFFBS method, respectively.

Algorithm D.1: MCMC algorithm for the semi-Markov model with SM-fullFFBS.

Initialise: Draw $\boldsymbol{\theta} \sim \pi(\boldsymbol{\theta})$ and generate $\mathbf{X}_{1:T}^{[1:C]} \sim \pi\left(\mathbf{X}_{1:T}^{[1:C]} \mid \boldsymbol{\theta}\right)$;

for $j = 1, 2, \ldots, J$ **do**

  Propose $\mathbf{X}_{1:T}^{[1:C]*} \sim \pi_H\left(\mathbf{X}_{1:T}^{[1:C]} \mid \mathbf{Y}_{1:T}^{[1:C]}, \kappa = 1, \boldsymbol{\theta}_{-\kappa}\right)$ with FFBS;

  Compute $a = \min\left(1, \frac{\pi_H\left(\mathbf{X}_{1:T}^{[1:C]} \mid \mathbf{Y}_{1:T}^{[1:C]}, \kappa=1, \boldsymbol{\theta}^{-\kappa}\right)}{\pi_H\left(\mathbf{X}_{1:T}^{[1:C]*} \mid \mathbf{Y}_{1:T}^{[1:C]}, \kappa=1, \boldsymbol{\theta}^{-\kappa}\right)} \times \frac{\pi\left(\mathbf{X}_{1:T}^{[1:C]*}, \boldsymbol{\theta} \mid \mathbf{Y}_{1:T}^{[1:C]}\right)}{\pi\left(\mathbf{X}_{1:T}^{[1:C]}, \boldsymbol{\theta} \mid \mathbf{Y}_{1:T}^{[1:C]}\right)}\right)$;

  Draw $u \sim$ Uniform(0,1);

  if $u \leq a$ **then**

    Set $\mathbf{X}_{1:T}^{[1:C]} = \mathbf{X}_{1:T}^{[1:C]*}$;

  else

    Set $\mathbf{X}_{1:T}^{[1:C]} = \mathbf{X}_{1:T}^{[1:C]}$;

  end

  Perform suitable MCMC update to sample $\boldsymbol{\theta} \sim \pi\left(\boldsymbol{\theta} \mid \mathbf{Y}_{1:T}^{[1:C]}, \mathbf{X}_{1:T}^{[1:C]}\right)$;

end

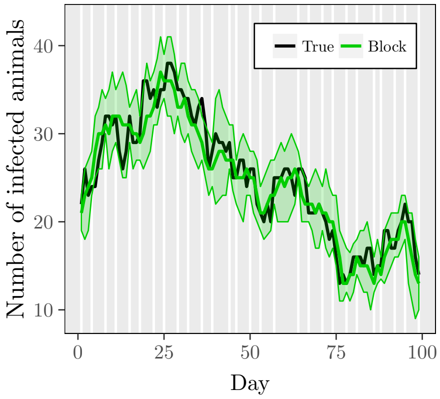

(a) Block updates method.

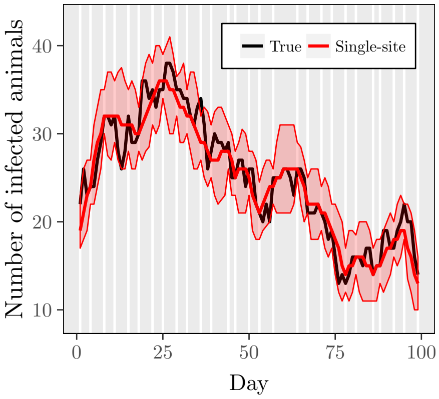

(b) Single-site update method.

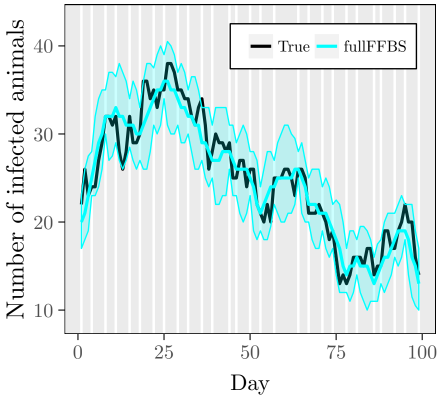

(c) fullFFBS method.

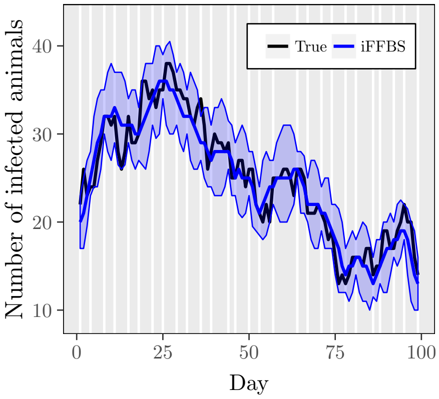

(d) iFFBS method.

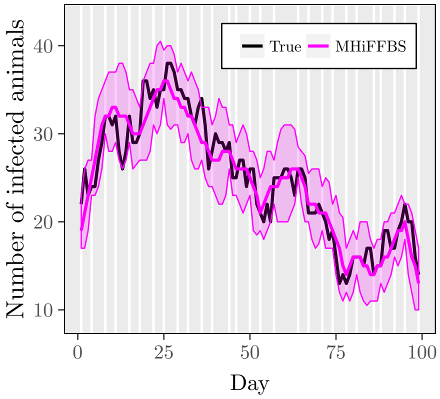

(e) MHiFFBS method.

Figure C.1: Posterior median number of infected individuals for the Markov model. Black solid lines represent the true values used to simulated the data. Shaded regions represent the 95% credible intervals. White vertical lines represent the days where samples were assumed to be collected.

Table C.1: Relative speed comparison of the five methods in the Markov model (a) as a function of the total number of cattle per pen C and (b) as a function of study period T. Bold entries represent the most efficient method in each setup.

(a) Varying number of animals over a 14-week period study.

**Number of animals**	**Methods**				
	**Block**	**Single-site**	**fullFFBS**	**iFFBS**	**MHiFFBS**
3	1.00	2.86	**23.75**	23.63	11.25
4	1.00	2.90	**26.56**	25.66	13.78
5	1.00	2.74	**25.70**	24.23	13.31
6	1.00	2.64	**22.69**	22.28	11.59
7	1.00	2.99	19.55	**23.97**	13.18
8	1.00	2.91	15.84	**22.03**	12.17
9	1.00	2.80	4.29	**20.90**	11.52
10	1.00	2.52	1.21	**28.50**	10.60
11	1.71	5.33	1.00	**49.87**	21.66

(b) Varying study period with 8 animals per pen.

**Study period**	**Methods**				
	**Block**	**Single-site**	**fullFFBS**	**iFFBS**	**MHiFFBS**
4 weeks	1.00	1.96	7.02	**9.18**	5.79
9 weeks	1.00	2.31	9.50	**11.74**	9.77
14 weeks	1.00	3.18	14.89	**21.79**	12.53
19 weeks	1.00	3.09	14.29	**22.37**	12.96
24 weeks	1.00	3.60	15.40	**19.88**	15.56
29 weeks	1.00	4.14	16.34	**21.74**	17.60
34 weeks	1.00	4.60	18.34	**25.15**	18.40
39 weeks	1.00	5.46	20.76	**28.26**	20.30
44 weeks	1.00	5.98	22.15	**31.28**	22.07

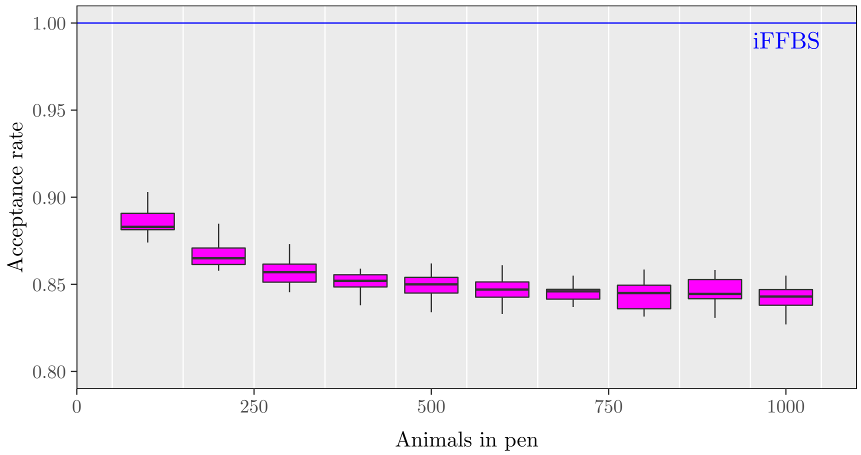

Figure C.2: Variability of the median acceptance rate of the MHiFFBS algorithm for the Markov model, based on 200 replications. The simulated data are generated with values of $C = 100, 200, \dots, 1000$. Blue horizontal line represent the acceptance rate of the iFFBS.

Algorithm D.2: MCMC algorithm for the semi-Markov model with SM-iFFBS.

Initialise: Draw $\boldsymbol{\theta} \sim \pi(\boldsymbol{\theta})$ and generate $\mathbf{X}^{[1:C]}_{1:T} \sim \pi\left(\mathbf{X}^{[1:C]}_{1:T} \mid \boldsymbol{\theta}\right)$;

for j = *1, 2, ..., J* **do**

  for c = *1, 2, ..., C* **do**

    Propose $\mathbf{X}^{[c]*}_{1:T} \sim \pi\left(\mathbf{X}^{[c]}_{1:T} \mid \mathbf{Y}^{[c]}_{1:T}, \mathbf{X}^{[-c]}_{1:T}, \kappa = 1, \boldsymbol{\theta}_{-\kappa}\right)$ with iFFBS;

    Compute $a = \min\left(1, \frac{\pi\left(\mathbf{X}^{[c]}_{1:T} \mid \mathbf{Y}^{[c]}_{1:T}, \mathbf{X}^{[-c]}_{1:T}, \kappa=1, \boldsymbol{\theta}^{-\kappa}\right)}{\pi\left(\mathbf{X}^{[c]*}_{1:T} \mid \mathbf{Y}^{[c]}_{1:T}, \mathbf{X}^{[-c]}_{1:T}, \kappa=1, \boldsymbol{\theta}^{-\kappa}\right)} \times \frac{\pi\left(\mathbf{X}^{[c]*}_{1:T}, \mathbf{X}^{[-c]}_{1:T}, \boldsymbol{\theta} \mid \mathbf{Y}^{[1:C]}_{1:T}\right)}{\pi\left(\mathbf{X}^{[c]}_{1:T}, \mathbf{X}^{[-c]}_{1:T}, \boldsymbol{\theta} \mid \mathbf{Y}^{[1:C]}_{1:T}\right)}\right)$;

    Draw $u \sim$ Uniform(0,1);

    if $u \leq a$ **then**

      Set $\mathbf{X}^{[c]}_{1:T} = \mathbf{X}^{[c]*}_{1:T}$;

    else

      Set $\mathbf{X}^{[c]}_{1:T} = \mathbf{X}^{[c]}_{1:T}$;

    end

  end

  Perform suitable MCMC update to sample $\boldsymbol{\theta} \sim \pi\left(\boldsymbol{\theta} \mid \mathbf{Y}^{[1:C]}_{1:T}, \mathbf{X}^{[1:C]}_{1:T}\right)$;

end

D.2 Updating the transmission parameters

The joint posterior distribution of the hidden infection states and the parameters is given by,

$$\pi(\mathbf{X}_{1:T}^{[1:C]}, \tilde{\alpha}, \tilde{\beta}, \tilde{m}, \kappa, \nu, \theta_R, \theta_F \mid \mathbf{Y}_{1:T}^{[1:C]}) \\ \propto \pi(\mathbf{Y}_{1:T}^{[1:C]} \mid \mathbf{X}_{1:T}^{[1:C]}, \theta_R, \theta_F)\ \pi(\mathbf{X}_{1:T}^{[1:C]} \mid \tilde{\alpha}, \tilde{\beta}, \tilde{m}, \kappa, \nu)\ \pi(\tilde{\boldsymbol{\theta}}), \qquad (1)$$

where $\pi(\tilde{\boldsymbol{\theta}})$ is the prior of the transformed model parameters $\tilde{\boldsymbol{\theta}} = (\tilde{\alpha}, \tilde{\beta}, \tilde{m}, \kappa, \nu, \theta_R, \theta_F)$, $\tilde{\alpha} = \log(\alpha)$, $\tilde{\beta} = \log(\beta)$ and $\tilde{m} = m - 1$. The prior distributions are specified exactly as in the Markov model for the parameters $\alpha, \beta, \tilde{m}, \nu, \theta_R$ and θ_F. We choose a Gamma prior for the additional parameter $\kappa \sim \text{Ga}(b_\kappa, c_\kappa)$.

The first term in Equation (1) can be written as,

$$\pi(\mathbf{Y}_{1:T}^{[1:C]} \mid \mathbf{X}_{1:T}^{[1:C]}, \theta_R, \theta_F) = \prod_{p=1}^{P}\prod_{c=1}^{C}\prod_{t=1}^{T} f_{x_t^{[c,p]}}\left(y_t^{[c,p]} \mid \theta_R, \theta_F\right),$$

and the second term is given by,

$$\pi(\mathbf{X}_{1:T}^{[1:C]} \mid \tilde{\alpha}, \tilde{\beta}, \tilde{m}, \kappa, \nu) = \prod_{p=1}^{P}\prod_{t=2}^{T}\left[\left(1 - \exp\left\{-e^{\tilde{\alpha}} - e^{\tilde{\beta}}\sum_{c=1}^{C} x_{t-1}^{[c,p]}\right\}\right)^{N_{01}^{p}(t)} \right. \\ \left. \times \left(\exp\left\{-e^{\tilde{\alpha}} - e^{\tilde{\beta}}\sum_{c=1}^{C} x_{t-1}^{[c,p]}\right\}\right)^{N_{00}^{p}(t)}\right] \times \prod_{p=1}^{P}\prod_{c=1}^{C}\left[\nu^{x_1^{[c,p]}}(1-\nu)^{1-x_1^{[c,p]}}\right] \\ \times \prod_{p=1}^{P}\prod_{c=1}^{C}\left[\left[\frac{1 - \sum_{\zeta=1}^{\zeta_1^{*[c,p]}-1}\mathbb{P}\big(Z=\zeta\big)}{\tilde{m}+1}\right]^{X_1^{[c,p]}}\left[\mathbb{P}\big(Z=\zeta_1^{*[c,p]}\big)\right]^{1-X_1^{[c,p]}}\right. \\ \left. \times \left[1 - \sum_{\zeta=1}^{\zeta_{\tau^{[c,p]}}^{*[c,p]}-1}\mathbb{P}\big(Z=\zeta\big)\right]^{X_{T^{[c,p]}}^{[c,p]}}\left[\mathbb{P}\big(Z=\zeta_{\tau^{[c,p]}}^{*[c,p]}\big)\right]^{1-X_{T^{[c,p]}}^{[c,p]}}\prod_{t=2}^{\tau^{[c,p]}-1}\mathbb{P}\big(Z=\zeta_t^{*[c,p]}\big)\right],$$

where $N_{0j}^{p}(t)$ denotes the number of individuals in pen p who were in state 0 at time $t-1$ and in state j at time t, for $j \in \{0,1\}$, $\left(\zeta_1^{*[c,p]}, \zeta_2^{*[c,p]}, \ldots, \zeta_{\tau^{[c,p]}}^{*[c,p]}\right)$ denotes the observed

infection durations vector for each individual c in pen p, and

$$\mathbb{P}(Z=\zeta)=\left(\frac{\kappa}{\kappa+\tilde{m}}\right)^{\kappa}\frac{\Gamma(\kappa+\zeta-1)}{(\zeta-1)!\,\Gamma(\kappa)}\left(\frac{\tilde{m}}{\kappa+\tilde{m}}\right)^{\zeta-1}.$$

The full conditional distribution of $\tilde{\alpha}$, $\tilde{\beta}$, $\tilde{m}$ and κ cannot be solved analytically and therefore we use HMC to update these parameters. We have that the partial derivatives are given by:

$$\frac{\partial\log\pi(\tilde{\alpha},\tilde{\beta},\tilde{m},\kappa\mid\cdot)}{\partial\tilde{\alpha}}=\sum_{p=1}^{P}\sum_{t=2}^{T}\left[N_{01}^{p}(t)\times e^{\tilde{\alpha}}\times\frac{\exp\left\{-e^{\tilde{\alpha}}-e^{\tilde{\beta}}\sum_{c=1}^{C}x_{t-1}^{[c,p]}\right\}}{1-\exp\left\{-e^{\tilde{\alpha}}-e^{\tilde{\beta}}\sum_{c=1}^{C}x_{t-1}^{[c,p]}\right\}}\right.$$

$$\left.-N_{00}^{p}(t)\times e^{\tilde{\alpha}}\right]+\frac{\partial\log\pi_{\alpha}(e^{\tilde{\alpha}})}{\partial\tilde{\alpha}}+1,$$

$$\frac{\partial\log\pi(\tilde{\alpha},\tilde{\beta},\tilde{m},\kappa\mid\cdot)}{\partial\tilde{\beta}}=\sum_{p=1}^{P}\sum_{t=2}^{T}\left[N_{01}^{p}(t)\times e^{\tilde{\beta}}\times\sum_{c=1}^{C}x_{t-1}^{[c,p]}\times\frac{\exp\left\{-e^{\tilde{\alpha}}-e^{\tilde{\beta}}\sum_{c=1}^{C}x_{t-1}^{[c,p]}\right\}}{1-\exp\left\{-e^{\tilde{\alpha}}-e^{\tilde{\beta}}\sum_{c=1}^{C}x_{t-1}^{[c,p]}\right\}}\right.$$

$$\left.-N_{00}^{p}(t)\times e^{\tilde{\beta}}\times\sum_{c=1}^{C}x_{t-1}^{[c,p]}\right]+\frac{\partial\log\pi_{\beta}(e^{\tilde{\beta}})}{\partial\tilde{\beta}}+1,$$

$$\frac{\partial\log\pi(\tilde{\alpha},\tilde{\beta},\tilde{m},\kappa\mid\cdot)}{\partial\tilde{m}}=\sum_{p=1}^{P}\sum_{c=1}^{C}\left[X_{1}^{[c,p]}\times\frac{-\sum_{\zeta=1}^{\zeta_{1}^{*\,[c,p]}-1}\frac{\partial\mathbb{P}(Z=\zeta)}{\partial\tilde{m}}}{1-\sum_{\zeta=1}^{\zeta_{1}^{*\,[c,p]}-1}\mathbb{P}(Z=\zeta)}-\frac{X_{1}^{[c,p]}}{\tilde{m}+1}\right.$$

$$+\frac{\left(1-X_{1}^{[c,p]}\right)}{\mathbb{P}\left(Z=\zeta_{1}^{*\,[c,p]}\right)}\times\frac{\partial\mathbb{P}\left(Z=\zeta_{1}^{*\,[c,p]}\right)}{\partial\tilde{m}}+\frac{-\sum_{\zeta=1}^{\zeta_{\tau^{[c,p]}}^{*\,[c,p]}-1}\frac{\partial\mathbb{P}(Z=\zeta)}{\partial\tilde{m}}}{1-\sum_{\zeta=1}^{\zeta_{\tau^{[c,p]}}^{*\,[c,p]}-1}\mathbb{P}(Z=\zeta)}$$

$$
+ \frac{\left(1 - X_{\tau^{[c,p]}}^{[c,p]}\right)}{\mathbb{P}\left(Z = \zeta_{\tau^{[c,p]}}^{*\,[c,p]}\right)} \times \frac{\partial \mathbb{P}\left(Z = \zeta_{\tau^{[c,p]}}^{*\,[c,p]}\right)}{\partial \tilde{m}} + \sum_{t=2}^{\tau^{[c,p]}-1} \frac{\frac{\partial \mathbb{P}\left(Z = \zeta_t^{*\,[c,p]}\right)}{\partial \tilde{m}}}{\mathbb{P}\left(Z = \zeta_t^{*\,[c,p]}\right)} \Bigg]
$$

$$
+ \frac{\partial \log \pi_{\tilde{m}}(\tilde{m})}{\partial \tilde{m}},
$$

$$
\frac{\partial \log \pi(\tilde{\alpha}, \tilde{\beta}, \tilde{m}, \kappa \mid \cdot)}{\partial \kappa} = \sum_{p=1}^{P} \sum_{c=1}^{C} \Bigg[X_1^{[c,p]} \times \frac{- \sum_{\zeta=1}^{\zeta_1^{*\,[c,p]}-1} \frac{\partial \mathbb{P}(Z = \zeta)}{\partial \kappa}}{1 - \sum_{\zeta=1}^{\zeta_1^{*\,[c,p]}-1} \mathbb{P}(Z = \zeta)}
$$

$$
+ \frac{\left(1 - X_1^{[c,p]}\right)}{\mathbb{P}\left(Z = \zeta_1^{*\,[c,p]}\right)} \times \frac{\partial \mathbb{P}\left(Z = \zeta_1^{*\,[c,p]}\right)}{\partial \kappa} + \frac{- \sum_{\zeta=1}^{\zeta_{\tau^{[c,p]}}^{*\,[c,p]}-1} \frac{\partial \mathbb{P}(Z = \zeta)}{\partial \kappa}}{1 - \sum_{\zeta=1}^{\zeta_{\tau^{[c,p]}}^{*\,[c,p]}-1} \mathbb{P}(Z = \zeta)}
$$

$$
+ \frac{\left(1 - X_{\tau^{[c,p]}}^{[c,p]}\right)}{\mathbb{P}\left(Z = \zeta_{\tau^{[c,p]}}^{*\,[c,p]}\right)} \times \frac{\partial \mathbb{P}\left(Z = \zeta_{\tau^{[c,p]}}^{*\,[c,p]}\right)}{\partial \kappa} + \sum_{t=2}^{\tau^{[c,p]}-1} \frac{\frac{\partial \mathbb{P}\left(Z = \zeta_t^{*\,[c,p]}\right)}{\partial \kappa}}{\mathbb{P}\left(Z = \zeta_t^{*\,[c,p]}\right)} \Bigg]
$$

$$
+ \frac{\partial \log \pi_{\kappa}(\kappa)}{\partial \kappa}.
$$

As before, we use a fixed number of leapfrog steps $L = 30$ and adopt the stepsize ϵ during burnin to obtain an acceptance rate of roughly 65% as suggested by Neal (2011).

E Additional results for the semi-Markov model

In this section we provide additional results for the analysis of the simulated data from the semi-Markov model of Section 4.3. The estimated number of infected individuals over time, along with 95% credible intervals, as obtained from one of the 200 runs for each of the five methods considered is shown in Figure E.1. Results of relative speed for several values of C and T are shown in Table E.1(a) and Table E.1(b) respectively.

Figure E.2 considers simulations with the number of individuals per pen vary between 100 and 1000, as obtained from 20 replicates. Figure E.3 shows the posterior summaries of parameter κ under twenty different scenarios, $\kappa = 0.5, 1, 1.5, \ldots, 10$. Notice that for large values of κ then SM-fullFFBS algorithm fails to infer the correct value of κ, due to poor mixing of the MCMC as a consequence of very low acceptance rates for the missing data, as shown in Figure E.4.

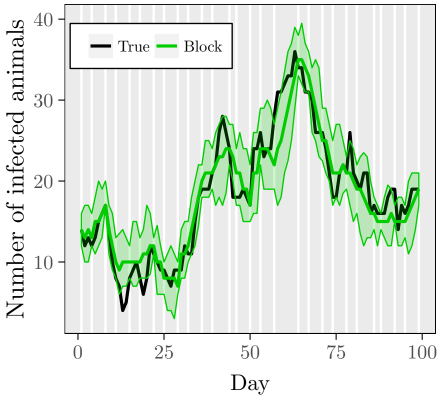

(a) Block updates method.

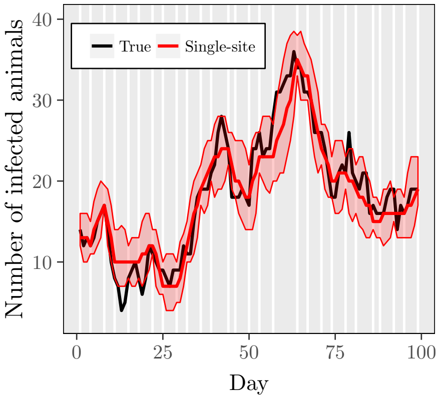

(b) Single-site update method.

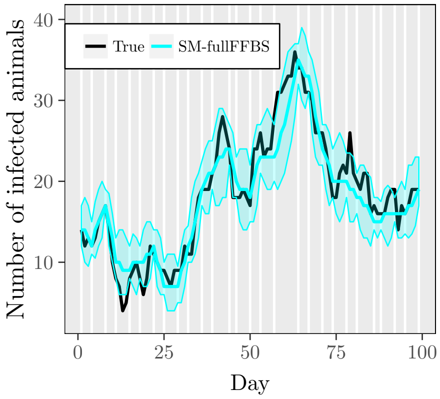

(c) SM-fullFFBS method.

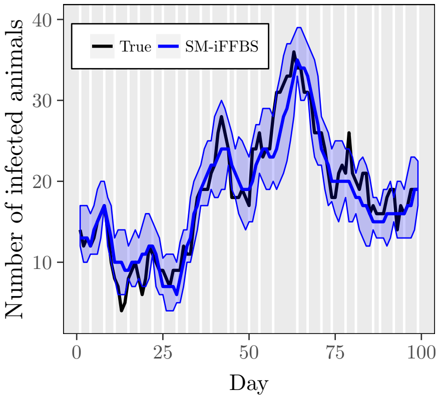

(d) SM-iFFBS method.

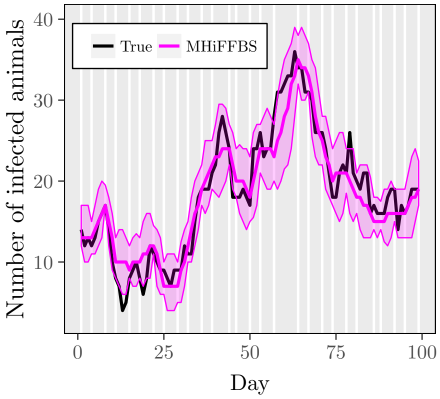

(e) MHiFFBS method.

Figure E.1: Median posterior number of infected individuals for the semi-Markov model. Black solid lines represent the true values used to simulated the data. Shaded regions represent the 95% credible intervals. White vertical lines represent the days where samples were collected.

Table E.1: Relative speed comparison of the five methods in the semi-Markov model (a) as a function of the total number of cattle per pen C and (b) as a function of study period T. Bold entries represent the most efficient method in each setup.

(a) Varying number of animals over a 14-week period study.

**Number of animals**	**Methods**				
	**Block**	**Single-site**	**SM-fullFFBS**	**SM-iFFBS**	**MHiFFBS**
3	1.00	1.13	**5.72**	4.34	4.12
4	1.22	1.00	5.42	7.49	**7.68**
5	1.12	1.00	6.69	8.31	**9.98**
6	1.22	1.00	5.99	7.49	**8.81**
7	1.36	1.00	5.40	10.38	**11.29**
8	1.41	1.00	4.63	10.48	**11.89**
9	1.48	1.17	1.00	10.56	**10.70**
10	5.97	3.91	1.00	21.85	**25.14**
11	19.99	13.33	1.00	**107.09**	98.96

(b) Varying study period with 8 animals per pen.

**Study period**	**Methods**				
	**Block**	**Single-site**	**SM-fullFFBS**	**SM-iFFBS**	**MHiFFBS**
4 weeks	1.30	1.00	1.26	2.72	**5.46**
9 weeks	1.82	1.00	2.33	8.69	**10.61**
14 weeks	1.38	1.00	3.58	10.34	**11.51**
19 weeks	1.26	1.00	2.64	10.08	**10.09**
24 weeks	1.45	1.00	2.37	12.82	**14.34**
29 weeks	1.78	1.00	3.10	10.16	**10.56**
34 weeks	1.24	1.00	2.25	9.45	**11.41**
39 weeks	1.21	1.00	1.84	10.98	**12.77**
44 weeks	1.36	1.00	2.35	**11.88**	10.83

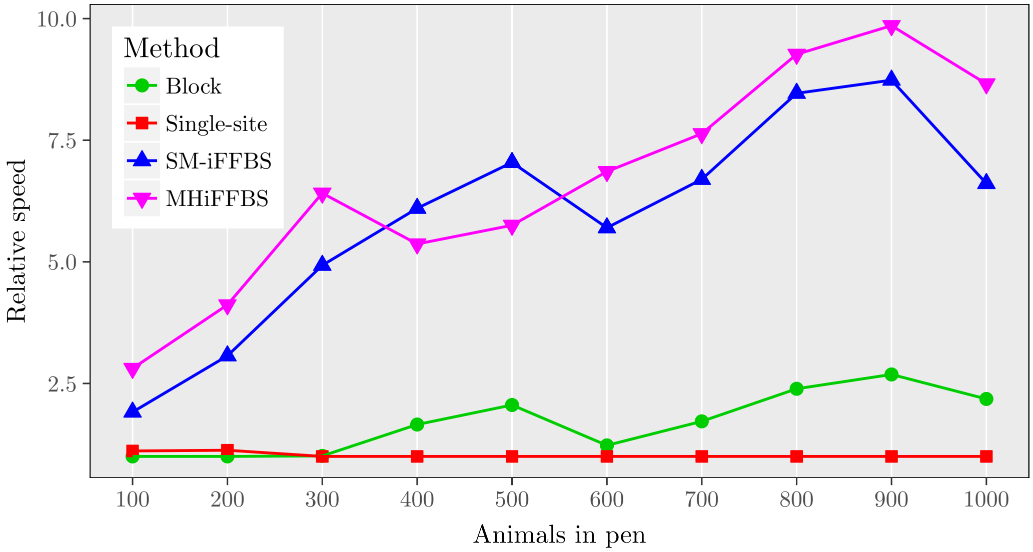

Figure E.2: Median relative speed comparison of four methods in the semi-Markov model for large datasets with values for $C = 100, 200, \ldots, 1000$, based on 20 simulations.

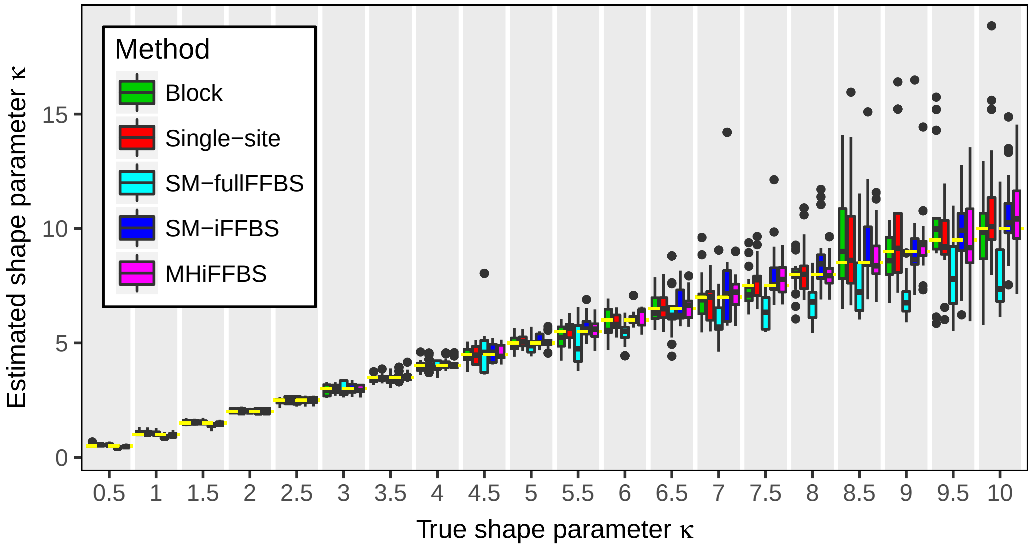

Figure E.3: Posterior summaries of parameter κ based on 200 replicates, under twenty different scenarios for semi-Markov model with 8 individuals. The simulated data are generated for values of $\kappa = 0.5, 1, 1.5, \ldots, 10$.

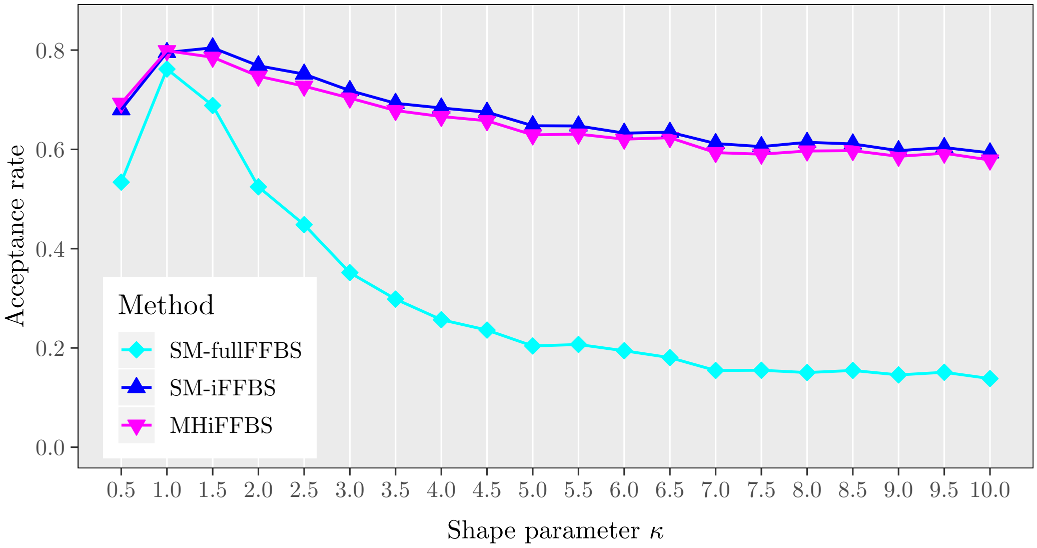

Figure E.4: Median acceptance rates of three methods based on 200 replicates, under different values of $\kappa = 0.5, 1, 1.5, \ldots, 10$ for semi-Markov model with 8 individuals.

F Application on real data

In this section we use the methods described throughout the main paper for the analysis of a real dataset concerning the transmission dynamics of *E. coli* O157:H7 in cattle presented in Section 4.1. We fit both the Markov and semi-Markov models. Priors specifications are identical to the ones used for the analysis of the simulated data in Sections 4.2 and 4.3. As before, we run the MCMC for 11,000 iterations, using the last 10,000 for calculating the relative efficiency. To get an estimate of the Monte Carlo variability of the efficiency measure we run 20 chains per method, with different starting values.

In terms of parameter estimation, posterior summaries of the model parameters are presented in Table F.1 where we observe that the estimates obtained from the different methods are almost identical. Note that we show the summaries only for the proposed iFFBS and MHiFFBS methods since the results obtained from the remaining methods are similar. Overall, our parameter estimates are in close agreement with results obtained by Spencer et al. (2015) who previously analysed the same data.

The comparison of computational efficiency yields conclusions which are analogous to the ones reached when comparing performance on simulated data. For the Markov model

we find a median relative speed of 1 for block proposals method, single-site method has 3.16, MHiFFBS has 14.65, while the fullFFBS and iFFBS are the most efficient with relative speeds of 18.96 and 24.27 respectively. For the semi-Markov the medians are 1 for single-site method, 1.33 for block updates, 3.37 for SM-fullFFBS, 7.87 for the SM-iFFBS and finally 8.65 for MHiFFBS. Results are shown in Figure F.1.

Table F.1: Posterior summaries for the parameters of both Markov and semi-Markov epidemic models, fit to the real *E. coli* O157:H7 dataset. The two methods presented are iFFBS and MHiFFBS. S.d. indicates standard deviation.

	**Geometric**				**Negative Binomial**			
**Symbol**	**iFFBS**		**MHiFFBS**		**SM-iFFBS**		**MHiFFBS**	
	**Mean**	**S.d.**	**Mean**	**S.d.**	**Mean**	**S.d.**	**Mean**	**S.d.**
α	0.009	0.001	0.009	0.001	0.008	0.001	0.008	0.001
β	0.011	0.002	0.011	0.002	0.010	0.002	0.010	0.002
m	9.365	0.743	9.227	0.789	10.033	0.837	10.061	0.822
κ	–	–	–	–	1.680	0.485	1.659	0.456
ν	0.098	0.025	0.099	0.025	0.099	0.026	0.098	0.025
θ_R	0.777	0.022	0.775	0.024	0.772	0.024	0.774	0.023
θ_F	0.466	0.022	0.464	0.022	0.462	0.023	0.464	0.022

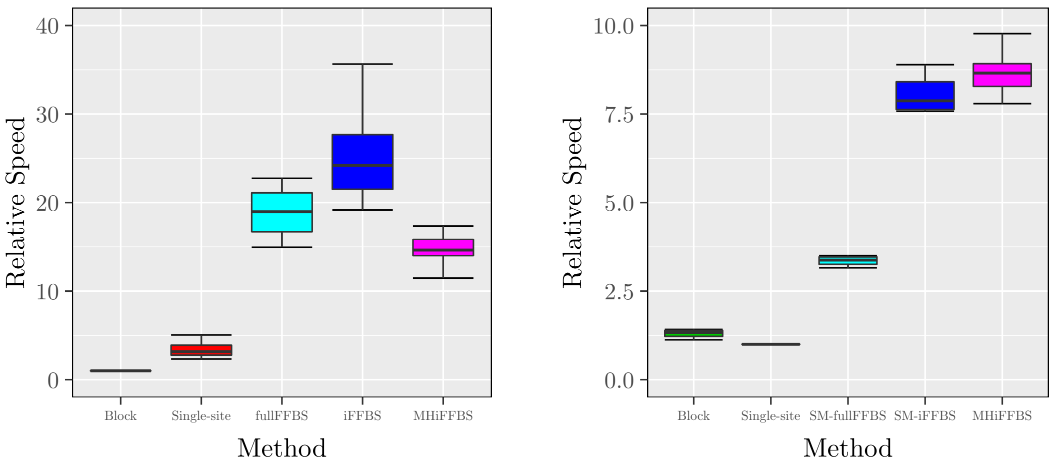

(a) Markov model.

(b) Semi-Markov model.

Figure F.1: Relative speed comparison of methods when applied for the analysis of the real *E. coli* O157:H7 dataset. (a) Results for the Markov model. (b) Results for the semi-Markov model. Quantiles are obtained from 20 different replicates.

References

Neal, R. M. (2011). MCMC using Hamiltonian dynamics. In S. Brooks, A. Gelman, G. L. Jones, and X. Meng (Eds.), *Handbook of Markov Chain Mote Carlo*, Chapter 5, pp. 113–162. Chapman & Hall/CRC.

Spencer, S. E. F., T. E. Besser, R. N. Cobbold, and N. P. French (2015). 'Super' or just 'above average'? Supershedders and the transmission of *Escherichia coli* O157:H7 among feedlot cattle. *Journal of The Royal Society Interface 12*(110), 20150446.
